# Specified data for tonsil surgery in Germany

**DOI:** 10.3205/cto000135

**Published:** 2016-12-15

**Authors:** Jochen P. Windfuhr

**Affiliations:** 1 Department of Otolaryngology, Head & Neck Surgery, Allergology, Kliniken Maria Hilf, Mönchengladbach, Germany

**Keywords:** tonsillectomy, tonsillotomy, surgical prevalence, bleeding complications, post-tonsillectomy hemorrhage, peritonsillar abscess, incisional drainage, abscess tonsillectomy

## Abstract

**Background:** Tonsillectomy rates vary considerably among different states, regions, and times. This study was conducted to identify the prevalence of “chronic” tonsillitis, peritonsillar abscess, hypertrophy of the tonsils with and without adenoids in absolute and relative numbers in an 80 million people nation. Moreover, the number and rates of different surgical procedures to resolve either “chronic” tonsillitis, peritonsillar abscess, or upper airway obstruction due to (adeno)tonsillar hypertrophy over several years was evaluated in this study (tonsillectomy, adenotonsillectomy, tonsillotomy, abscess tonsillectomy, transoral incision and drainage). Finally, the post-tonsillectomy hemorrhage rate was calculated and analyzed in relation to age and gender.

**Material and methods:** Calculations were based on data as published by the Federal Institute of Statistics or on request, if needed. The latest data were provided for 2013.

**Results:** The total number of the aforementioned diseases (stratified by ICD-10) decreased from 142,574 (in 2000) to 87,624 in 2013 (38.5%). Tonsillectomy, with or without adenoidectomy, was performed in a total of 833,896 patients between 2006 and 2013 in Germany. The yearly number decreased continually from 120,993 in 2006 to 84,332 procedures in 2013 (30.3%). The most significant decrease was registered in patients younger than 20 years of age for this time period: 70.92 per 10,000 in 2010 to 58.68 per 10,000 in 2013. If all age groups were included, the rate decreased from 13.34 per 10,000 to 10.90 per 10,000. In contrast, an increasing number of tonsillotomies was observed between 2007 (4,659 procedures) and 2013 (11,493). The cumulated number of procedures was 59,049. A constant number of 15,000 cases with peritonsillar abscess were diagnosed per year in Germany (19 patients per 100,000). The prevalence increased significantly at an age of 15 years and there was a preponderance of female patients below that age. Compared to the transoral incision and drainage, a 2.8-fold greater number of abscess tonsillectomies were performed annually. Post-tonsillectomy hemorrhage was experienced in 5.98% of all patients after 245,721 procedures in 2010 and 2013 (all indications, except tonsillotomy). Bleeding complications had occurred less frequently in female patients (5.06% vs. 7.02%). Finally, a considerable increase of post-tonsillectomy hemorrhage in patients older than 10 years of age was registered in male patients only.

**Conclusion:** Chronic tonsillitis was less frequently diagnosed and surgically treated in terms of tonsillectomy (with or without adenoidectomy), particularly in female patients. In contrast, the number of tonsillotomies increased continually, particularly in male patients. Peritonsillar abscess was diagnosed and surgically treated in a constant number of patients in the yearly comparison. Most of these patients were scheduled for abscess tonsillectomy, and only a 2.8-fold smaller number for transoral incision and drainage. Independent from the indication for surgery, post-tonsillectomy hemorrhage was clearly associated with male gender and age (>10 years). The study reveals a dramatic change mandating further surveillance in insurance companies and authorities in the national health system of an 80 million people nation. (Tab. 1)

## 1 Introduction

In 2004, Van Den Akker et al. [1] reported of different tonsillectomy rates in different countries and in the time course. The rate varied between 19 (Canada) and 118 (Ireland) per 10,000 children as well as 19 (Canada) and 76 (Finland) per 10,000 adolescents. In the Netherlands, the rate decreased from 290 in 1974 to 96 in 1985 per 10,000 children, and between 1985 and 1995 it increased from 20 to 43 per 10,000 adolescents. In Great Britain, the rate had increased from 50 in 1980ies to 80 in the 1990ies per 10,000 children and from 22 in 1980 to 30 in 1995 per 10,000 adolescents [1]. The different frequency was notified on a national and regional basis of different countries [2], [3], [4], [5], [6], [7], [8], [9], [10], [11] and during time periods of the same region [11], [12]. Concerning tonsillectomy (TE), age-related differences are accompanied by increasing rates of tonsillotomy (TOTO) – at least in Sweden, Austria, and Germany – [10], [13], [14].

The present study aimed to answer the following questions for Germany:

- How many patients were treated for chronic tonsillitis, tonsillar hypertrophy, and adenotonsillar hypertrophy on an inpatient basis?
- How many patients were scheduled for TE and TOTO per year? What are the rates per 100,000 inhabitants?
- What is the rate of post-tonsillectomy hemorrhage? Are age and gender risk factors for post-tonsillectomy hemorrhage?
- How many patients were treated for peritonsillar abscess (PTA) and many patients underwent surgery (abscesstonsillectomy or transoral incisional drainage)?

## 2 Material and methods

The data material of the Federal Statistical Office (Statistisches Bundesamt) in Wiesbaden, Germany, regarding number of inhabitants, frequencies, and rates of different surgical procedures is provided on one hand by online researches, e.g. databases of “Surgeries and procedures of hospitalized patients of hospitals” (Operationen und Prozeduren der vollstationären Patientinnen und Patienten der Krankenhäuser) [15], [16], [17], [18], [19], [20]. On the other hand, individually ordered evaluations based on the 6-digit OPS code were used. The Federal Statistical Office provides a variety of tables with the possibility to change variables like diagnosis, gender, years and so on [21]. The changes can be seen immediately online, but the results cannot be cited as an URL in this paper due to their temporary character.

Comparable registers for outpatient procedures, such as TOTO, do not exist. The performance of “tonsillotomy” is not part of the official outpatient performance catalogue (annex 2 of the Physicians’ Fee Scale; Einheitlicher Bewertungsmaßstab, EBM). This catalogue dictates which kind of procedure has to be performed on an outpatient basis. Unfortunately, these procedures have a different code and they are not reported to the Federal Statistical Office. Instead, there are special TOTO contracts and thus a large number of regionally agreed scales of fees where one cannot be sure what the service exactly is or which position corresponds to which performance. Furthermore, the exact scope of the agreements is not known [22].

Several calculations with regard to the total population in Germany were based on the data published by the Federal Statistical Office that are listed in a table considering partly the age structure (Table 1 (Tab. 1)) [23].

## 3 Results

### 3.1 Prevalence

The most recent data on patients treated on an inpatient basis were issued by the Federal Statistical Office in 2013. In this particular year, a total of 578,440 inpatients were treated for ENT-specific diseases [24]. 

#### 3.1.1 Diagnosis of “chronic tonsillitis” (ICD-10: J35.0), age distribution [21]

Since 2006, the diagnostic data published by the Federal Statistical Office are available as yearly PDF files. These files list the numbers of cases of each diagnosis in total and stratified by age groups. Unfortunately, the diagnoses are only listed according to the 3-digit ICD-10 code. That means, that diseases of the tonsils are only differentiated as *acute tonsillitis* (ICD-10: J03), *peritonsillar abscess* (ICD-10: J36), or *chronic diseases of the palatal and pharyngeal tonsil* (ICD-10: J35). The latter is very unspecific and summarizes different diagnoses such as *chronic tonsillitis, hypertrophy of the palatal tonsils with* and *without hypertrophy of the pharyngeal tonsil, hypertrophy of the pharyngeal tonsil* as well as *other chronic diseases of the palatal and pharyngeal tonsil*. We will show later, that these entities are related to age. With time, the diagnostic frequency clearly decreased for the age groups of 1–5 years (2010–2013) and 15–20 years (2008–2013).

#### 3.1.2 Diagnosis of “chronic diseases of the palatal and pharyngeal tonsils” (ICD-10: J35), age distribution [21]

**Figure 1:**
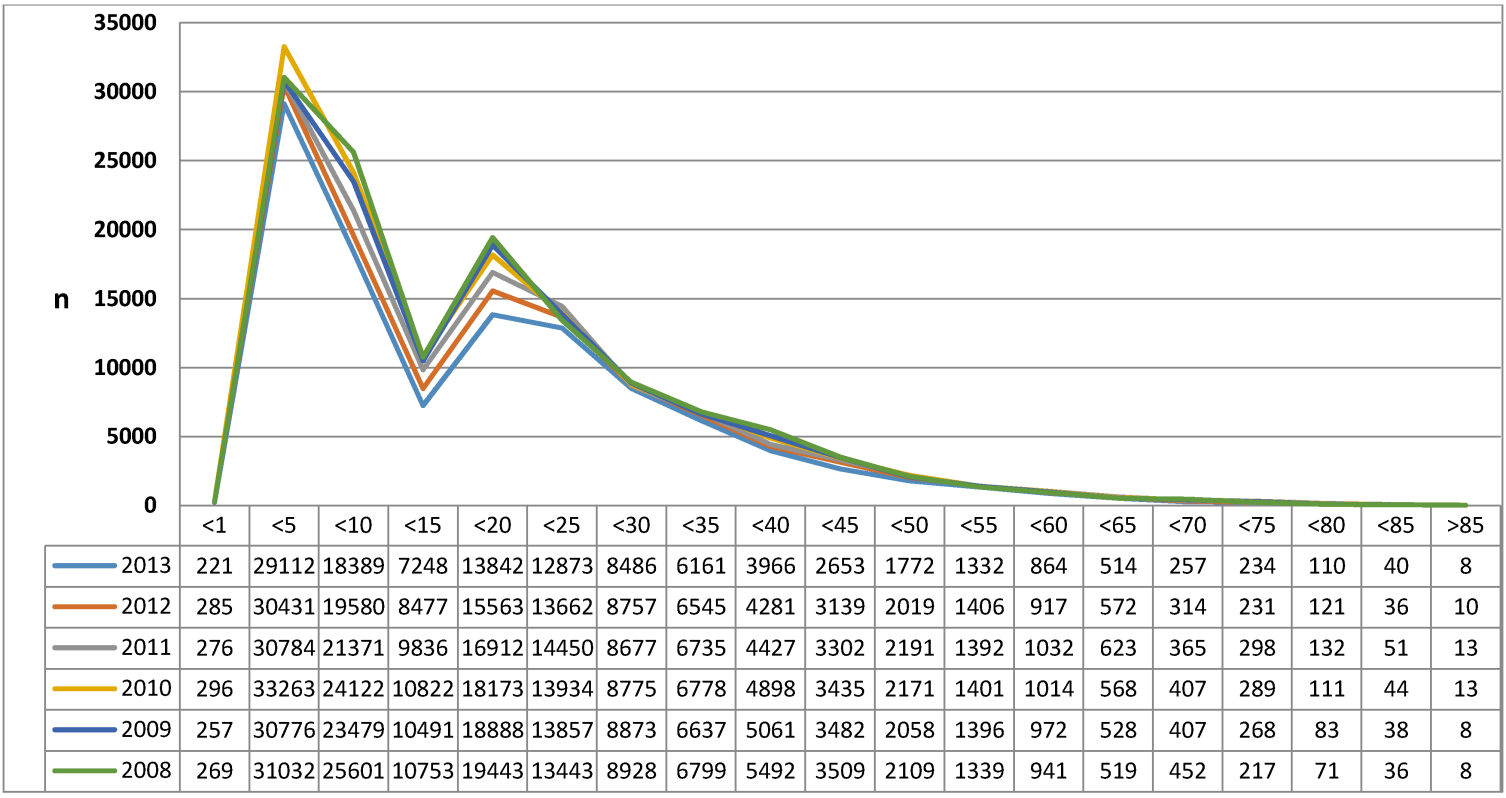
Diagnosis of “Chronic diseases of the palatal and pharyngeal tonsil” (ICD-10: J35) Diagram of the age groups in 5-year intervals with y-axis indicating the prevalence and x-axis the age group per year. On annual comparison, a decrease of the frequency of diagnosis in younger patients (<20 years) can be observed.

A differentiation of the single diagnoses of chronic tonsillitis, hypertrophy of the palatal tonsils with and without hypertrophy of the pharyngeal tonsil, hypertrophy of the pharyngeal tonsil as well as other chronic diseases of the palatal and pharyngeal tonsils based on the 4-digit ICD-10 code is described in Figure 1 (Fig. 1).

#### 3.1.3 Diagnosis of “chronic diseases of the palatal and pharyngeal tonsils” (ICD-10: J35), gender distribution [21]

**Figure 2:**
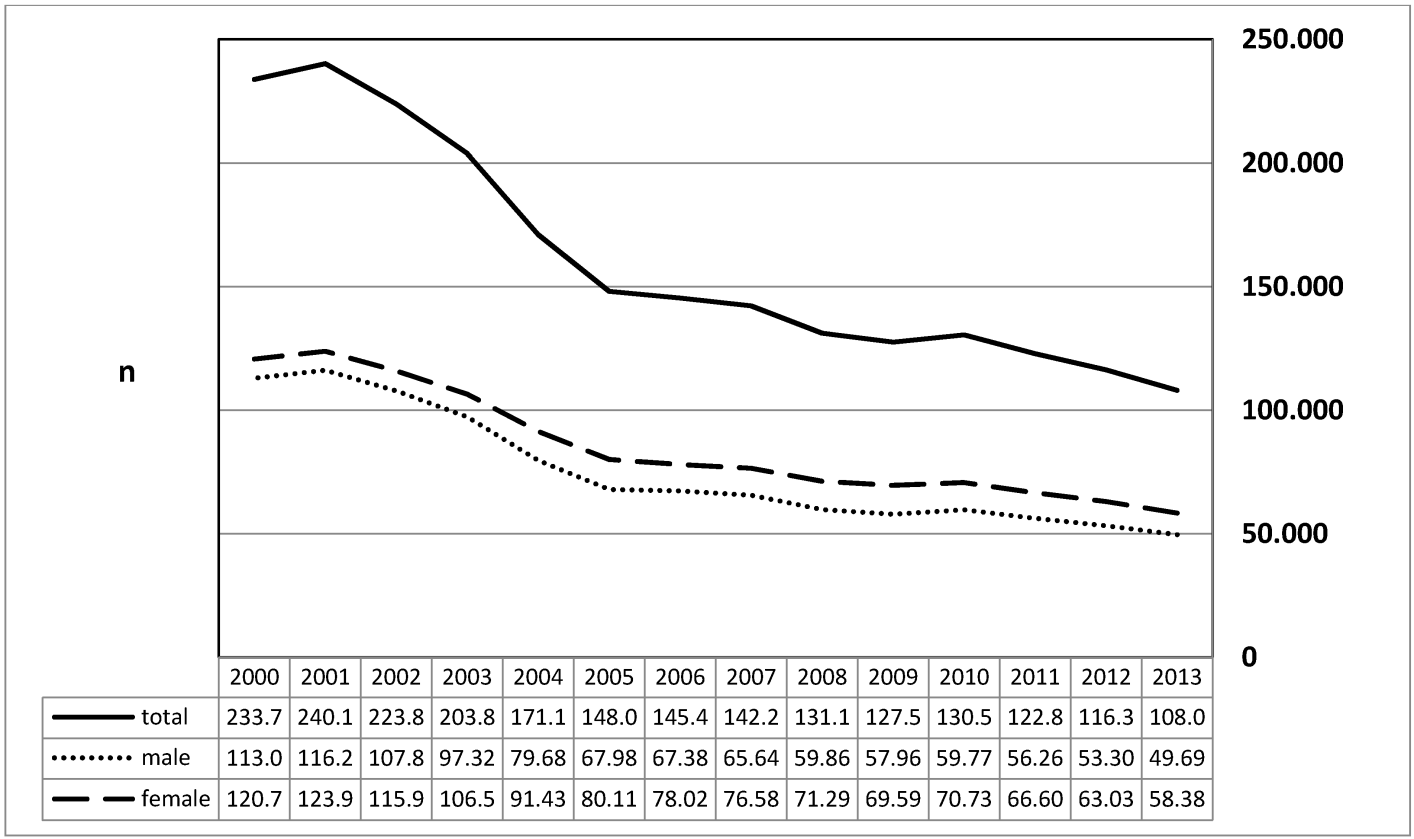
Diagnosis of “Chronic diseases of the palatal and pharyngeal tonsil” (ICD-10: J35) The y-axis indicates the prevalence on an annual basis for male and female patients (x-axis). A decreasing prevalence of the diagnosis is revealed for the time between 2000 and 2013 without gender preference (factor 2.16).

The graphic depiction shows the absolute numbers of female and male inpatients that decreased between 2000 and 2013 by factor 2.16. The data were taken from a table of the website of the Federal Statistical Office. The frequencies did not differ between female and male patients. Within the evaluation period, 1 to 18 deaths had occurred (mean: 6.9; median: 6.5). The number of short hospital stays (1–3 days) ranged between 38,906 and 62,859 (mean: 47,700; median: 44,905). The inpatient stay varied between 4 and 4.7 days (mean: 4.33; median 4.3 days) (Figure 2 (Fig. 2)).

#### 3.1.4 Diagnosis of “chronic tonsillitis” (ICD-10: J35.0)

**Figure 3:**
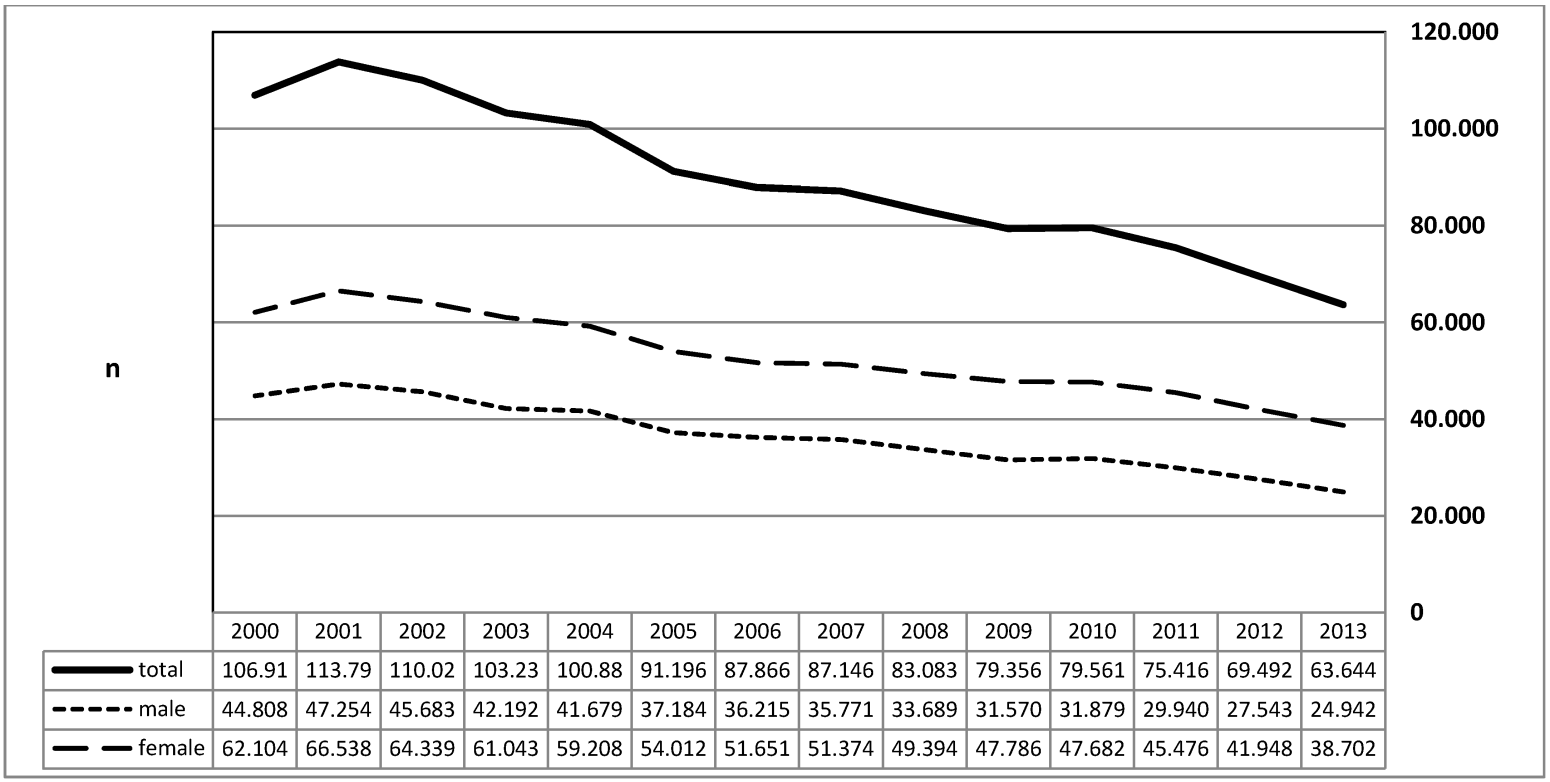
Diagnosis of “chronic tonsillitis” (ICD-10: J35.0). The graphs indicate the prevalence (y-axis) on a yearly basis for male and female patients (x-axis). Between 2000 and 2013, the number of these diagnoses decreased by factor 1.68. This decrease was registered more often in male patients (44.4%) than in female patients (37.7%).

Comparing the years of 2000 and 2013, the number of this diagnosis decreased by factor 1.68 (total: 40.5%; male patients: 44.4%; female patients: 37.7%). During the same time, the average time of inpatient stay decreased from 5.9 to 5.1 days. The number of days for calculation and hospitalization was reduced from 627,058 to 321,697 and the number of short hospital stays (1–3 days) increased from 8,069 to 12,136. During the entire period, there was female preponderance and an equal reduction in male and female patients. In each of the years of 2000, 2004, 2008, and 2009, one death was registered (Figure 3 (Fig. 3)).

#### 3.1.5 Diagnosis of “hypertrophy of the palatal tonsils” (ICD-10: J35.1) [21]

**Figure 4:**
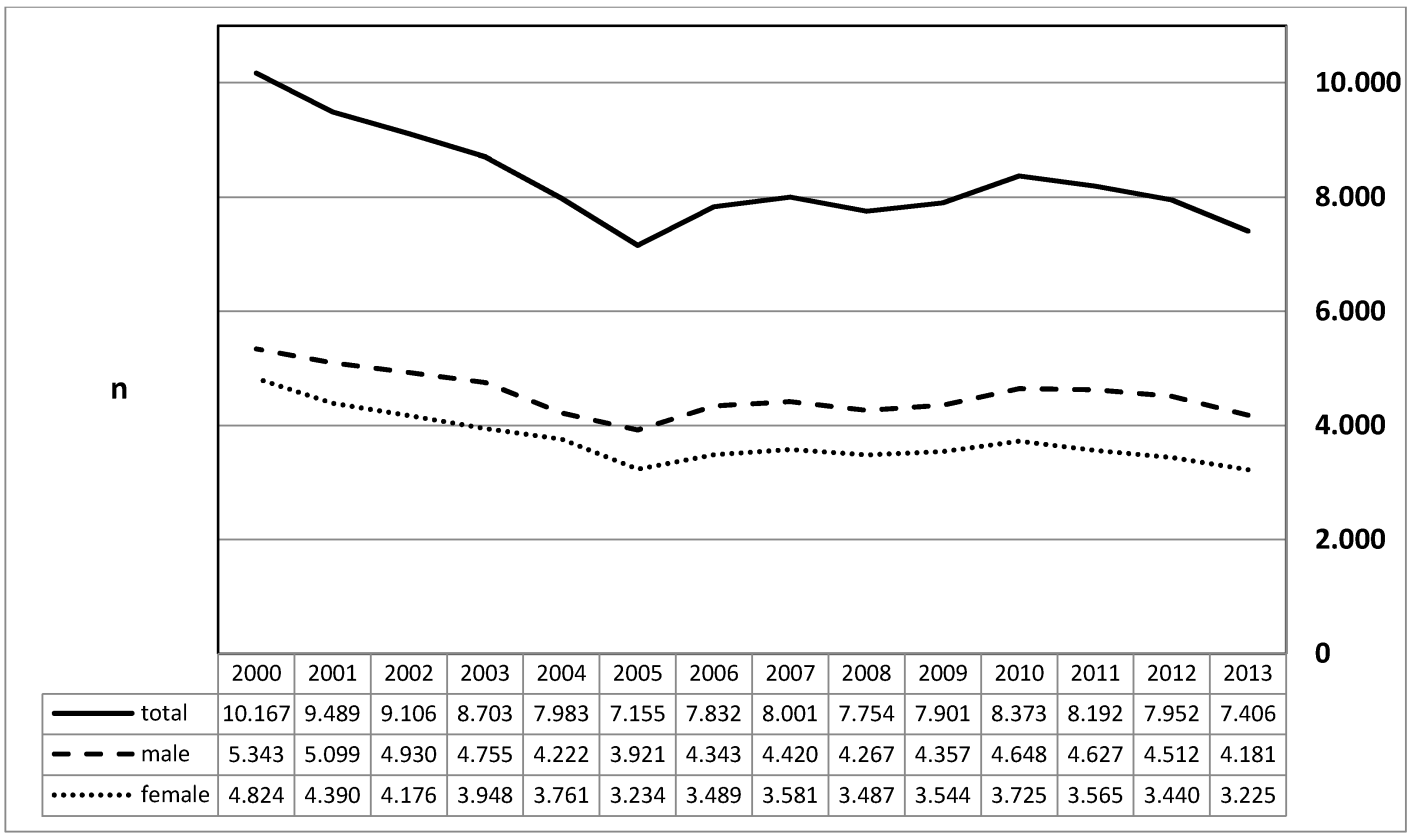
Diagnosis of “Hypertrophy of the palatal tonsils” (ICD-10: J35.1) The graphs indicated the annual prevalence (y-axis) for male and female patients (x-axis). Between 2000 and 2013, the number of cases had decreased by factor 1.37. Female and male patients were equally affected.

For this diagnosis, the comparison of 2000 and 2013 revealed a reduction of the number of inpatients by factor 1.37. The difference between male and female patients was not significant- in contrast to “chronic tonsillitis”. The decrease of the diagnostic frequency affected both, male and female, equally. In 2004 and 2013, one death was registered in each year. In the evaluation period, the average number of inpatient stays had decreased from 3.8 to 3.2 days while the number of short hospital stays (1–3 days) had increased from 2,638 to 4,757 cases (Figure 4 (Fig. 4)). 

#### 3.1.6 Diagnosis of “hypertrophy of the palatal tonsils with hypertrophy of the pharyngeal tonsils” (ICD-10: J35.3) [21]

**Figure 5:**
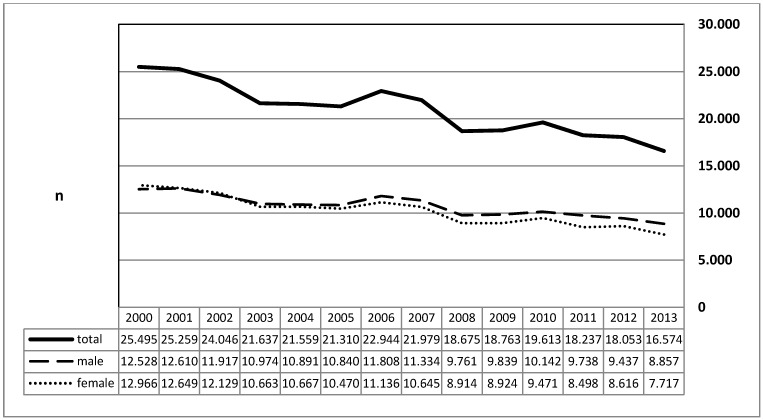
Diagnosis of “Hypertrophy of the palatal tonsils with hypertrophy of the pharyngeal tonsil” (ICD-10: J35.3) The graphs indicate the annual prevalence (y-axis) for male and female patients (x-axis). Between 2000 and 2013, the diagnostic frequency decreased by factor 1.53. The numbers for male and female patients were nearly identical.

The number of cases decreased between 2000 and 2013 (factor 1.53) with nearly identical numbers of female and male patients. No deaths were registered. The duration of inpatient stays decreased from 5.2 to 3.4, the number of short hospital stays (1–3 days) increased from 3,314 to 9,837 (Figure 5 (Fig. 5)).

### 3.2 Tonsillectomy

#### 3.2.1 Prevalence in Germany (OPS code: 5-281) [21]

**Figure 6:**
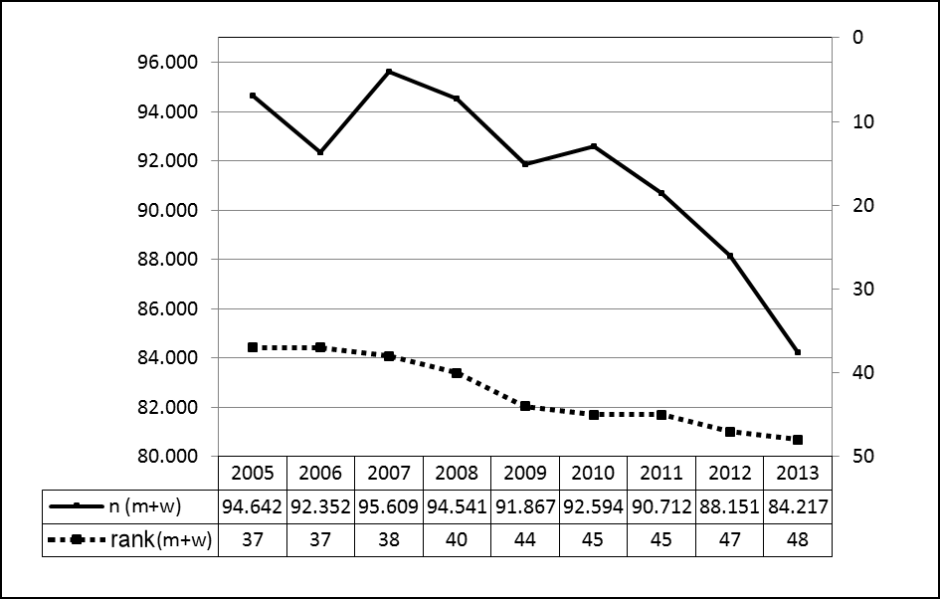
Tonsillectomy. Prevalence in Germany (OPS code 5-281) The Federal Statistical Office lists every year the 50 most frequent inpatient interventions in Germany since 2005. Among this group, TE without AT is regularly listed. Between 2005 and 2013, the total number of TE (without AT) decreased by 10,425 interventions (11%) and the surgery ranked 48 among the 50 most frequent interventions (rank 37 in 2005). The diagram shows the number of cases on the left y axis. On the right side the rank among the 50 most frequently performed procedures is depicted as indicated between 2005 and 2013 (x-axis). m = male; w = female

Since 2005, the Federal Statistical Office lists the 50 most frequent inpatient interventions in Germany on a yearly basis, always including TE without adenoidectomy (AT) [21]. The number of all 50 interventions totaled 6,227,289 in 2005 and 7,664,823 in 2013. Between 2005 and 2013, the number of TE was reduced by 10,425 (11%) and the intervention ranked 48 (previously 37) among the Top 50. It should be noted that the 4-digit OPS code (5-281 includes an array of interventions such as abscesstonsillectomy, revision procedures, radical and transpharyngeal TE as well as TOTO (on an inpatient basis). The absolute numbers and the ranking among the 50 most frequent inpatient procedures in Germany can be seen in a designable table (Figure 6 (Fig. 6); number of procedures vs. ranking among the 50 most frequent procedures per year).

##### 3.2.1.1 Tonsillectomy without adenoidectomy (OPS code: 5-281.0) – prevalence between 2005 and 2013

The numbers based on the 5-digit OPS code were taken from an evaluation of the Federal Statistical Office on request by the author. Between 2005 and 2013, a total of 627,874 interventions were performed, the annual number of cases had decreased by 24.2% (Table 2 (Tab. 2)).

##### 3.2.1.2 Tonsillectomy (OPS code: 5-281) stratified by age (2006–2013)

**Figure 7:**
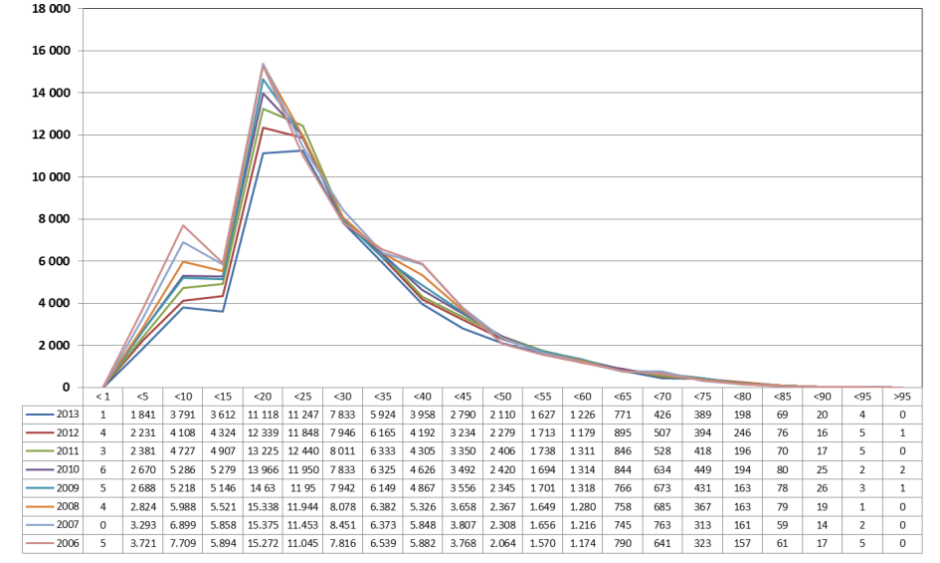
Tonsillectomy (OPS: 5-281), stratified based on the patients’ ages between 2006 and 2013 The graphs indicate the prevalence (y-axis) between 2006 and 2013 (x-axis), stratified by age groups (x-axis). A significant decrease was registered in younger patients (<20 years).

The numbers per age group were taken from material published by the Federal Statistical Office. Since 2006, all interventions are listed based on the 4-digit OPS code. The most recent data were available for 2013. After stratification into age groups, a significant decrease was observed in patients younger than 20 years between 2006 and 2013 (Figure 7 (Fig. 7)).

#### 3.2.2 Tonsillectomy (OPS code: 5-281), male and female patients

##### 3.2.2.1 Prevalence in male patients between 2005 and 2013 [21]

**Figure 8:**
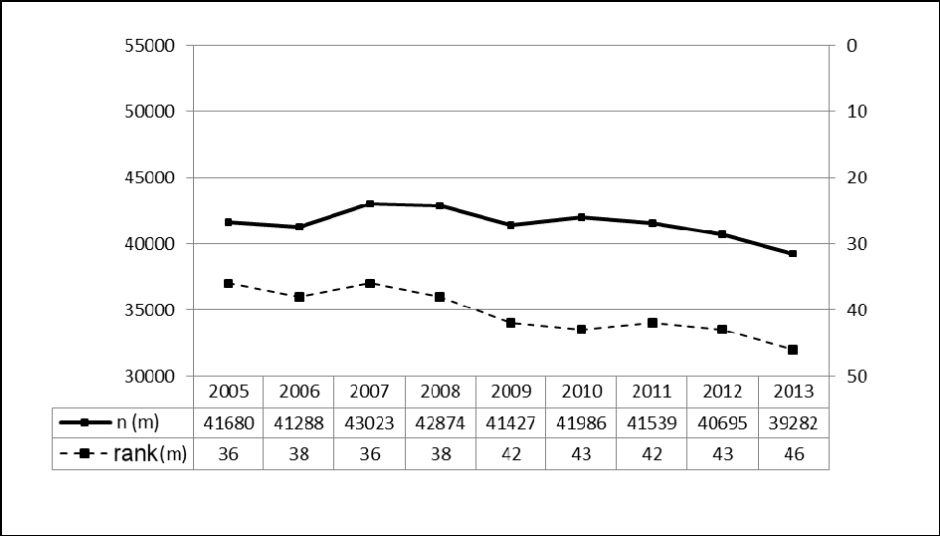
Tonsillectomy (OPS: 5-281), prevalence in male patients (2005-2013) The graphs indicate the prevalence in male patients (left y- axis) and the ranking within the 50 most common inpatient procedures (right y-axis) from 2005 through 2013 (x-axis). There was only a slight decrease of the prevalence in Germany. m = male

There was only a slight tendency of a decreasing frequency and ranking among the 50 most frequent interventions in male patients. It is therefore very likely, that the decreasing total frequency resulted from the group of female patients (Figure 8 (Fig. 8)).

##### 3.2.2.2 Prevalence in female patients between 2005 and 2013 (OPS code: 5-281)

**Figure 9:**
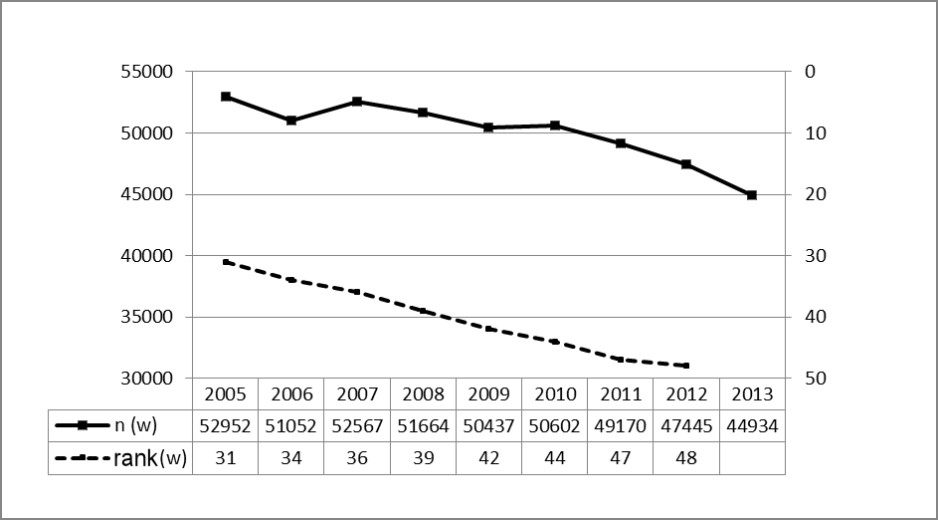
Tonsillectomy (OPS code: 5-281), prevalence in female patients (2005–2013) The graphs indicate the prevalence in male patients (left y-axis) and the ranking within the 50 most common inpatient procedures (right y-axis) from 2005 through 2013 (x-axis). The decrease of the prevalence was remarkable. In 2013, TE is no longer listed among the 50 most common inpatient procedures. The prevalence is higher compared to to male patients. w = female

A significant decrease in the numbers of procedures and ranking was registered in female patients since 2005. However, the number of female patients exceeded the one of male patients every year (Figure 9 (Fig. 9)).

##### 3.2.2.3 Male and female patients, stratified by age (OPS: 5-281.0)

The total number of tonsillectomies performed between 2005 and 2013 was different (17%) in female and male patients (450,823 vs. 373,794). Several evaluations of the Federal Statistical Office for 2010 and 2013 resulted on request by the author. The calculations were based on the 6-digit OPS code. The analysis revealed a clear decrease in the number of procedures in female patients and in patients between the 15 to 30 years of age.

##### 3.2.2.3.1 Tonsillectomy (OPS code: 5-281.0) in 2010, stratified by age

**Figure 10:**
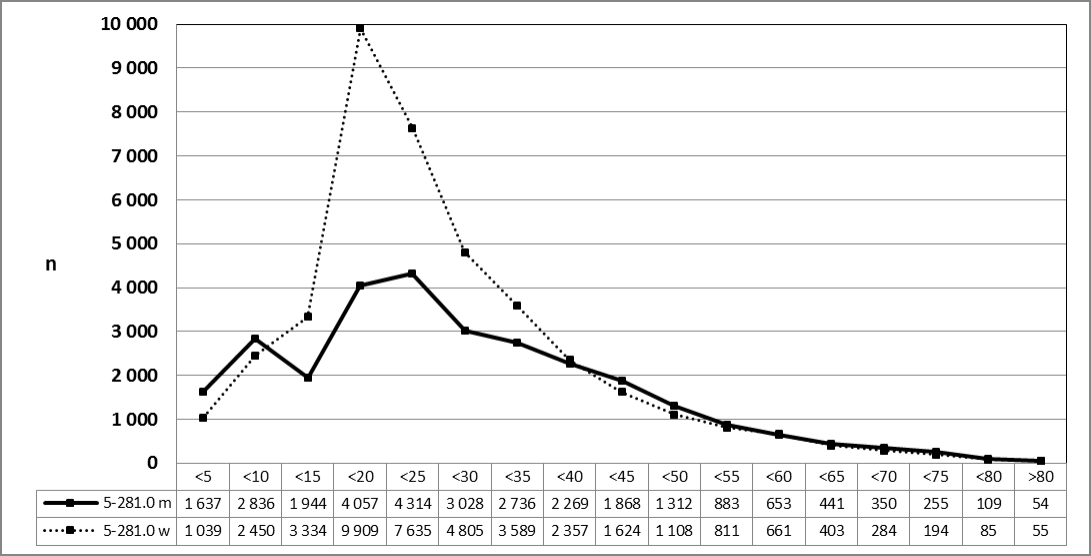
Tonsillectomy (OPS code: 5-281.0), prevalence in 2010, stratified by age and gender The graphs of male and female patients indicate the prevalence (y-axis) according to age groups (x-axis). There was a significant difference between male and female patients at the age of 15 to 30 years. m = male; w = female

See Figure 10 (Fig. 10).

##### 3.2.2.3.2 Tonsillectomy (OPS code 5-281.0) in 2013, stratified by age

**Figure 11:**
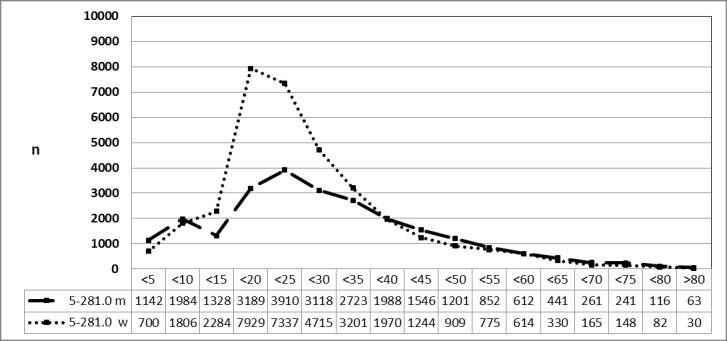
Tonsillectomy (OPS code: 5-281.0), prevalence in 2013, stratified by age and gender The graphs of male and female patients indicate the prevalence (y-axis) according age groups (x-axis). Comparable to 2010, a significant difference between male and female patients was found in the age 15 and 30 years. m = male; w = female

See Figure 11 (Fig. 11).

#### 3.2.3 Rate of tonsillectomy in younger patients (<20 years)

The evaluations for 2010 and 2013 enabled the author to calculate the rate of TE for patients <20 years of age due to the available population numbers in Germany, as published by the Federal Statistical Office [23]. Obviously, more female patients had undergone TE in this age group. The absolute number of interventions had decreased by 19.6% and the rate by 17.3% within 3 years (Table 3 (Tab. 3) and Table 4 (Tab. 4)).

### 3.3 Adenotonsillectomy (ATE)

#### 3.3.1 Number of procedures between 2006 and 2013

The numbers of this intervention are obtainable from publications of the Federal Statistical Office since 2006, based on the 4-digit OPS code [15], [16], [17], [18], [19], [20]. The most recent data were available for 2013. Within the evaluation period, a total of 283,787 interventions had been performed (Table 5 (Tab. 5)).

#### 3.3.2 Adenotonsillectomy (OPS code: 5-282), stratified by age, between 2006 and 2013

**Figure 12:**
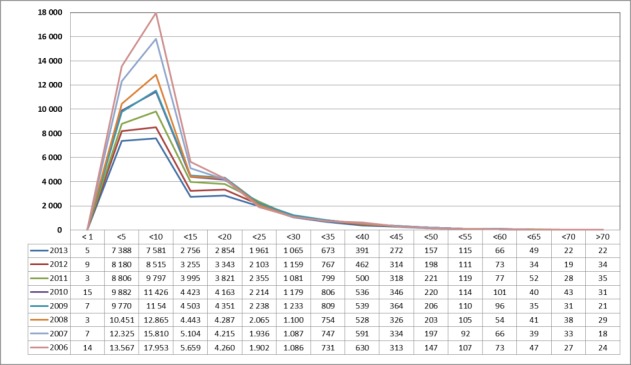
Adenotonsillectomy (OPS code: 5-282), prevalence between 2006 and 2013 The graphs indicate the prevalence (y-axis) between 2006 and 2013 (x-axis), stratified by age groups (x-axis). A significant decrease was registered in younger patients (<15 years).

The yearly number of procedures was retrieved from the Federal Statistical Office database, based on the 4-digit OPS code with most recent data available for 2013. After stratification into age groups, a significant reduction was obvious for the group of patients younger than 15 years (Figure 12 (Fig. 12)).

#### 3.3.3 Male and female patients, 2010 vs. 2013 (OPS code: 5-282.0)

**Figure 13:**
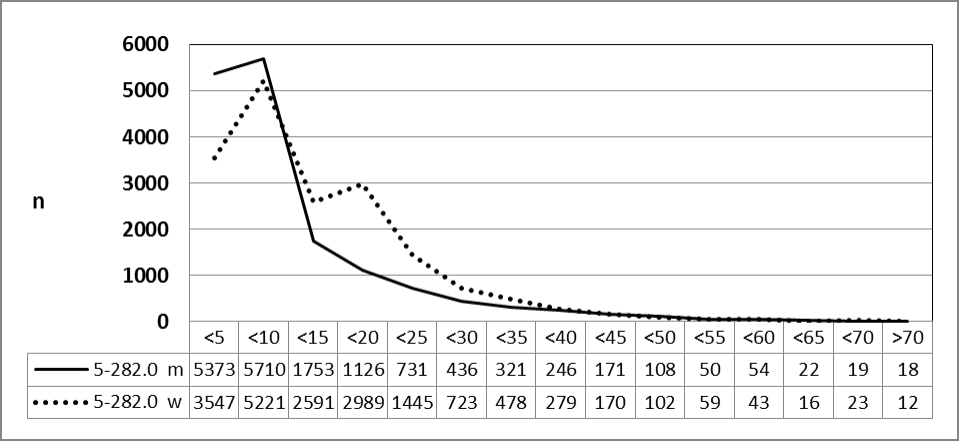
Adenotonsillectomy (OPS code: 5-282.0), frequency in 2010, detailed analysis The graphs of male and female patients indicate the prevalence (y-axis) according age groups (x-axis). A difference was only found for very young patients (<5 years) and adolescents (15–20 years). The prevalence decreased constantly with ages without gender preference. m = male; w = female

**Figure 14:**
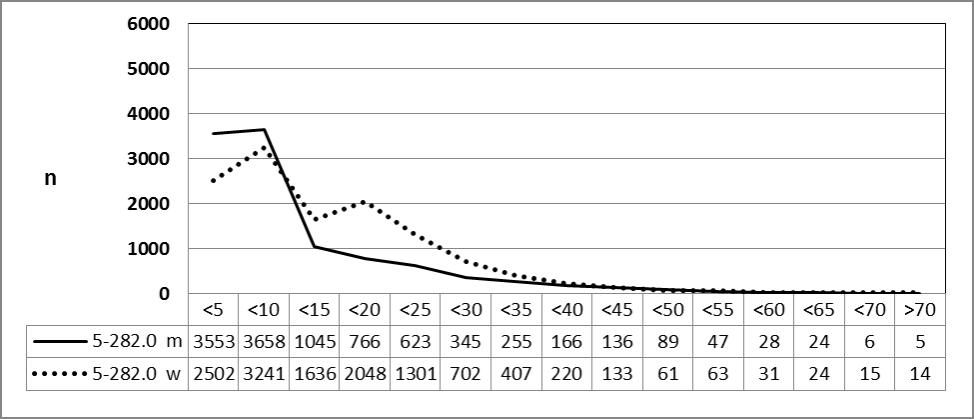
Adenotonsillectomy (OPS code: 5-282.0), frequency in 2013, detailed analysis The graphs of male and female patients indicate the prevalence (y-axis) according age groups (x-axis). A difference was only found for very young patients (<5 years) and adolescents (15–20 years). The prevalence decreased constantly with ages without gender preference. m = male; w = female

Special evaluations of the Federal Statistical Office for 2010 (above) and 2013 (below) on request by the authors revealed a significant decrease for male and female patients with a constant gender-specific frequency of procedures in both years (Figure 13 (Fig. 13) and Figure 14 (Fig. 14)). 

### 3.4 Tonsillotomy

#### 3.4.1 Prevalence since 2007

**Figure 15:**
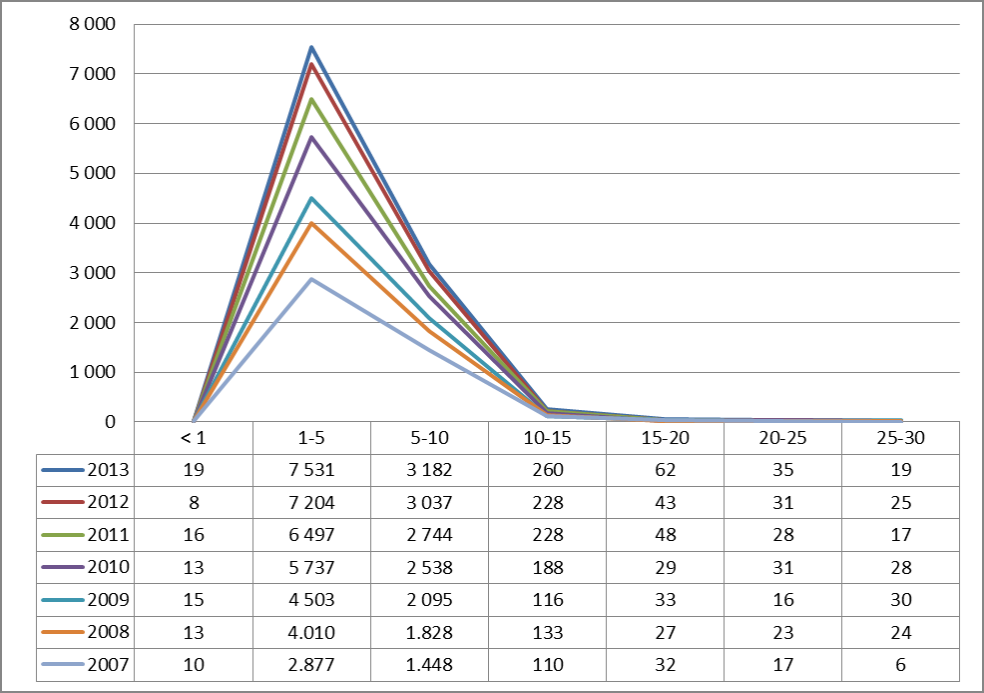
Tonsillotomy (OPS code: 5-281.5), prevalence. The graphs indicate the prevalence (y-axis) between 2007 and 2013 (x-axis), stratified by age groups (x-axis). Since this surgery is performed only rarelyabove the age of 15, not all age groups are depicted. The prevalence constantly increases in the group of 1 to 5 year-old patients since 2007.

A specific OPS-Code for TOTO was introduced in 2007 (5-281.5). Therefore, comparisons are with other inpatient procedures are only possible since 2007. The procedure is commonly performed in patients younger than 15 years of age and illustration therefore limited by age (≤30 years). The number of TOTOs multiplied by 2.61 within 6 years (Figure 15 (Fig. 15)). 

#### 3.4.2 Number of cases in 2010 and 2013 stratified by age and gender

**Figure 16:**
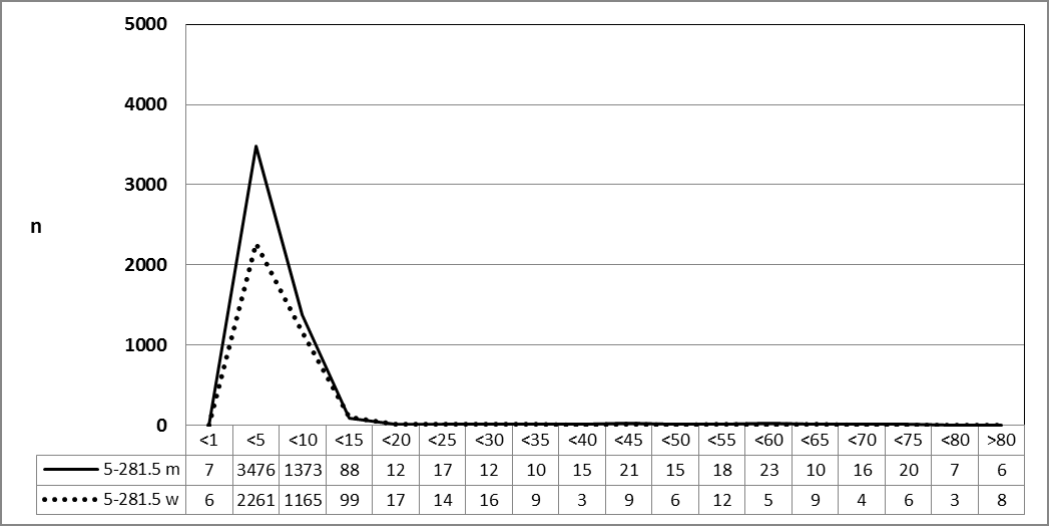
Tonsillotomy (OPS code: 5-281.5), prevalence in 2010, detailed analysis The graphs of male and female patients indicate the prevalence (y-axis) according age groups (x-axis). The intervention was almost only performed in younger patients (5 to 10 years), predominantly of male gender. m = male; w = female

**Figure 17:**
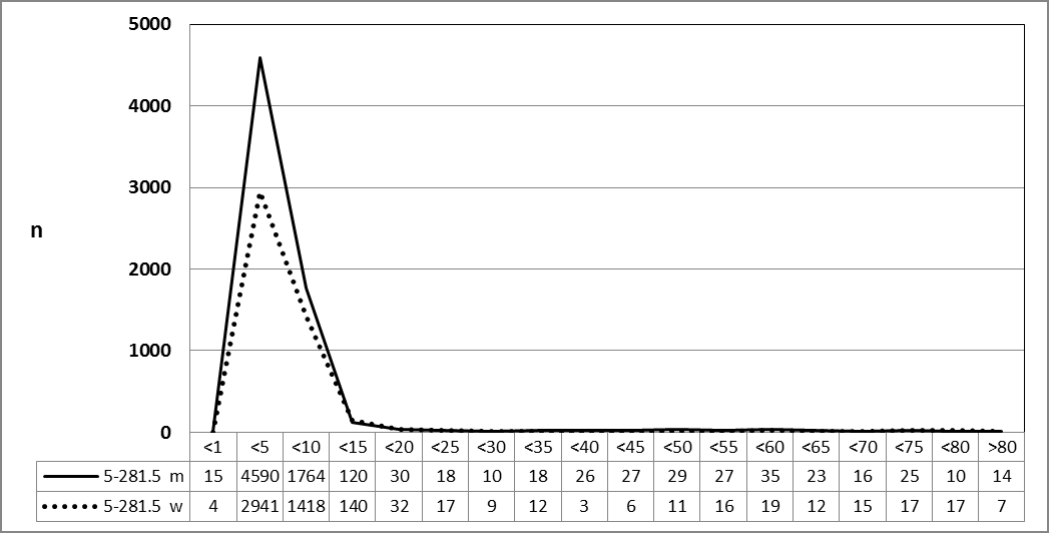
Tonsillotomy (OPS code: 5-281.5), frequency in 2013, detailed analysis The graphs of male and female patients indicate the prevalence (y-axis) according age groups (x-axis). The intervention was almost only performed in younger patients (5 to 10 years), predominantly of male gender. In comparison to 2010, 30.6% more patients had undergone TOTO. m = male; w = female

A specific database of the Federal Statistical Office was provided on request to stratify the data material for 2010 (n=8,798) and 2013 (n=11,493) by age and gender the gender. It is noteworthy to repeat, that the number of TOTs performed on an outpatient basis is not included. The number of TOTOs increased by 30.6% during the evaluation period and was almost exclusively performed in patients between 5 to 10 years of age. Male gender prevailed as for young patients (5 to 10 years of age) scheduled for TE (5-281.0) or ATE (5-282.0) (Figure 16 (Fig. 16) and Figure 17 (Fig. 17)).

#### 3.4.3 Prevalence of tonsillotomies (OPS code: 5-282.5) vs. tonsillectomies (OPS code: 5-281.0) since 2005

**Figure 18:**
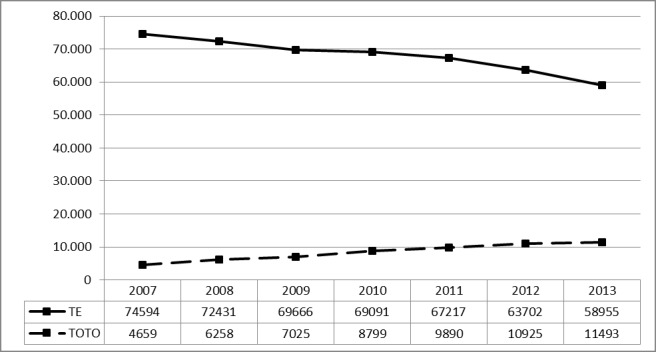
Prevalence of TOTO (OPS code: 5-281.5) vs. TE (OPS code: 5-281.0) The graphs indicate the prevalence (y-axis) between 2007 and 2013 (x-axis), stratified the type of intervention (TOTO vs. TE). The prevalence of TE decreased by 21%, but the prevalence of TOTO multiplied only by 2.5. However, the total number of interventions is decreasing with time.

On request of the authors, the Federal Statistical Office provided a database to compare the number of TE vs. TOTO, the number of ATE was not included (OPS code: 5-282.0). While the number of procedures of TE decreased by 21% during the evaluation period, the number of TOTO multiplied by 2.5. The annual sum of both interventions, however, decreased. It remains unclear, whether or not the number of outpatient TOTOs may compensate the decrease (Figure 18 (Fig. 18)).

### 3.5 Peritonsillar abscess (PTA)

#### 3.5.1 Development of the number of cases, rates, and health economics

The prevalence varies between 17.94 and 19.6 per 100,000 inhabitants (Table 6 (Tab. 6)) which is always less than the sum of abscesstonsillectomy and incisional drainage on a yearly analysis. This might result from a coding problem: peritonsillitis and PTA are coded as J36. It is also possible that alternative procedures such as interval tonsillectomy or needle aspiration or antibiotic alone were applied for a number of cases (Table 6 (Tab. 6)).

According to the data of the Federal Statistical Office, a total of 8,846 male and 6,576 female patients were treated for 5.0 days on average in 2013, including 4,134 short-term hospitalizations, 100 short-stay cases. There were 7 cases with lethal outcome. For 15,301 patients treated in 2013, an age-specific case number of 9 (<15 years), 34 (15–45 years), 13 (45–65 years), and 8 (>65 years) can be calculated per 100,000 inhabitants. The age-specific standardized case number in 2013 was approximately 19 per 100,000 inhabitants, for males a value of 16 per 100,000 inhabitants was mentioned (standard population “Germany 2011”) [21].

The data illustrate the economic burden of disease with yearly costs of approximately 30 million Euro. For the USA, roughly fivefold higher costs have been calculated [25]. Furthermore, the expenses must be added that result from absences from work. Databases with access options, however, are not established. The Federal Statistical Office, however, provided the data of AOK health insurance members (without pensioners) in form of a designable table as a source of information [21]. Thus, patients with PTA are absent from work for about 12 days (Table 7 (Tab. 7)).

#### 3.5.2 Incisional drainage vs. abscess tonsillectomy

**Figure 19:**
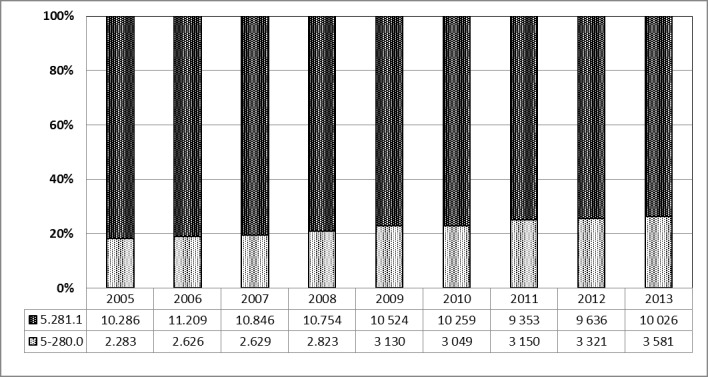
Prevalence of incisional drainage (OPS code: 5-280.0) *vs.* abscesstonsillectomy (OPS code: 5-281.1), 2005–2013 The columns depict the percentage (y-axis) of both interventions for each year (x-axis). Between 2005 and 2013, 2,955 incisional drainages and 10,026 abscess TEs on average were performed per year. The comparison of the data over 9 years reveals an increasing prevalence of transoral incisional drainage vs. abscess TE.

Between 2005 and 2013, 2,955 incisional drainages (median: 3,049±401.07) and 10,026 abscesstonsillectomy (median: 10,286±590.68) were performed per year on average. A comparison of the numbers over 9 years illustrates the increasing significance of transoral incisional drainage (OPS code: 5-280.0) vs. abscesstonsillectomy (OPS code: 5-281.1) (Figure 19 (Fig. 19)).

#### 3.5.3 Abscesstonsillectomy (OPS code: 5-281.1); prevalence between 2005 and 2013 with age distribution

**Figure 20:**
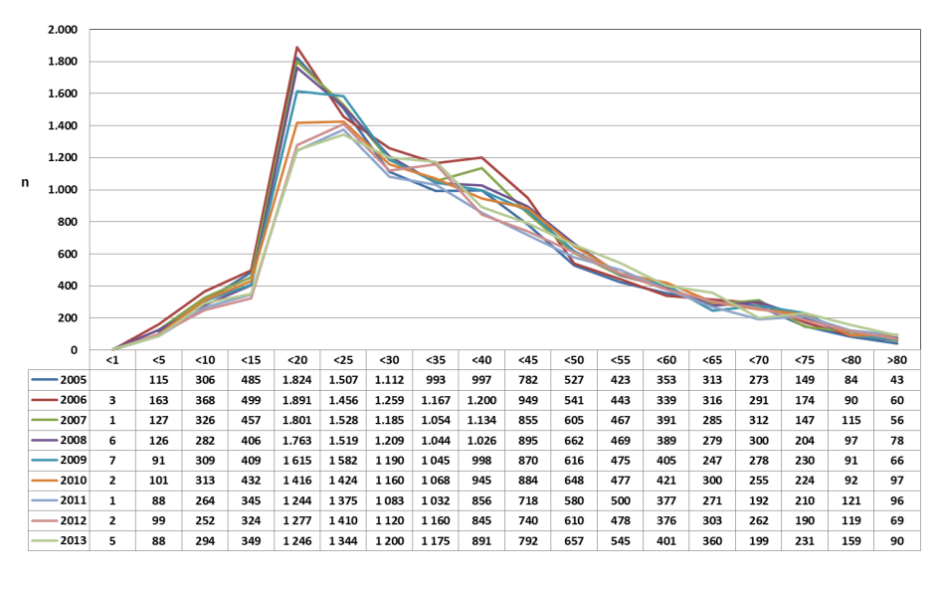
Abscess tonsillectomy (OPS code: 5-281.1); stratified based on the patients’ ages (2005–2013) The graphs indicate the prevalence (y-axis) between 2005 and 2013 (x-axis), stratified by age groups (x-axis). It can be noticed that most interventions were performed in adolescents and young adults (15 to 25 years of age) and a decreasing number of cases within the evaluation period in this age group. The prevalence increases abruptly after the age of 15 and is continually decreasing after the age of 25. The prevalence differs significantly to the prevalence of the diagnosis “chronic diseases of the palatal and pharyngeal tonsil”.

The database of the Federal Statistical Office was provided on request of the author to evaluate the yearly prevalence of abscesstonsillectomy within 2005 to 2013 without stratification by gender (Figure 20 (Fig. 20)). Some facts are remarkable:

- Highest frequency between the ages of 15 and 25 (risk group)
- Decreasing prevalence within the evaluation period in the risk group
- Sudden increase of the prevalence in patients older than 15 years of age
- Continuously decreasing frequency in patients older than 25 years of age
- Age-specific prevalence different to “chronic diseases of the palatal and pharyngeal tonsils” (ICD-10: J35)

#### 3.5.4 Abscesstonsillectomy, male vs. female patients

##### 3.5.4.1 2010 [26]

**Figure 21:**
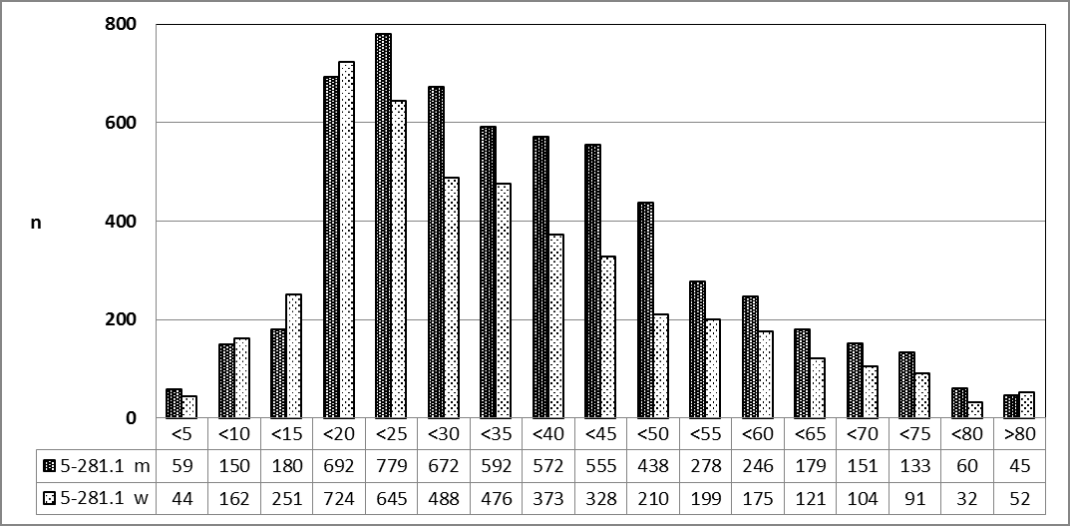
Abscess tonsillectomy (OPS code: 5-281.1), prevalence in 2010, detailed analysis The graphs of male and female patients indicate the prevalence (y-axis) according age groups (x-axis). Before the age of 20, mostly female patients were affected, afterwards mostly male patients. m = male; w = female

A specified analysis on request by the author [26] confirmed the finding, that patients younger than 20 years of age were mostly female and patients older than 20 years of age were male (Figure 21 (Fig. 21)).

##### 3.5.4.2 2013 [26]

**Figure 22:**
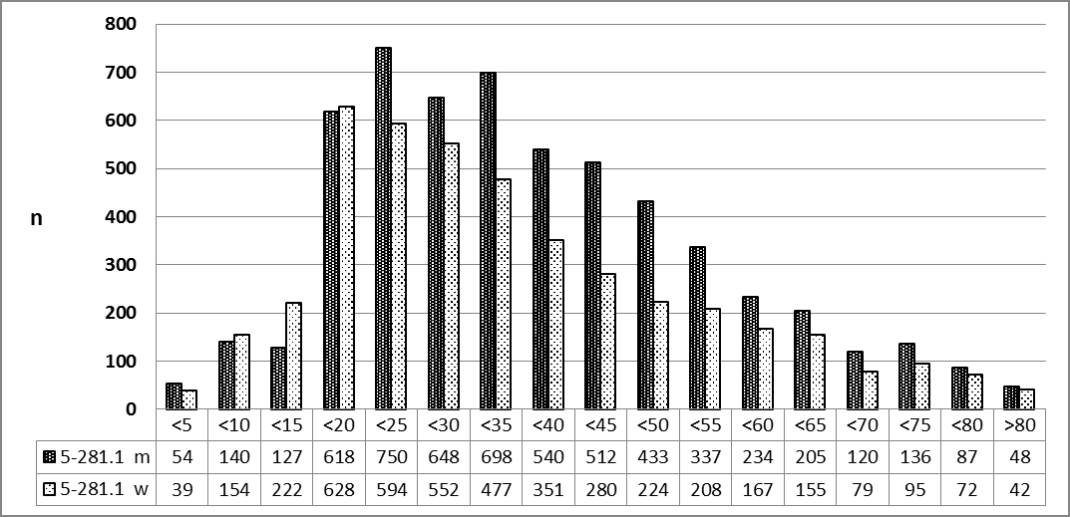
Abscess tonsillectomy (OPS code: 5-281.1), prevalence in 2013, detailed analysis The graphs of male and female patients indicate the prevalence (y-axis) according to age groups (x-axis). Before the age of 20, mostly female patients underwent surgery, afterwards male patients. m = male; w = female

The phenomenon mentioned in 3.5.4.1 is confirmed for 2013 (Figure 22 (Fig. 22)).

#### 3.5.5 Incisional drainage, male vs. female patients

##### 3.5.5.1 2010 [26]

**Figure 23:**
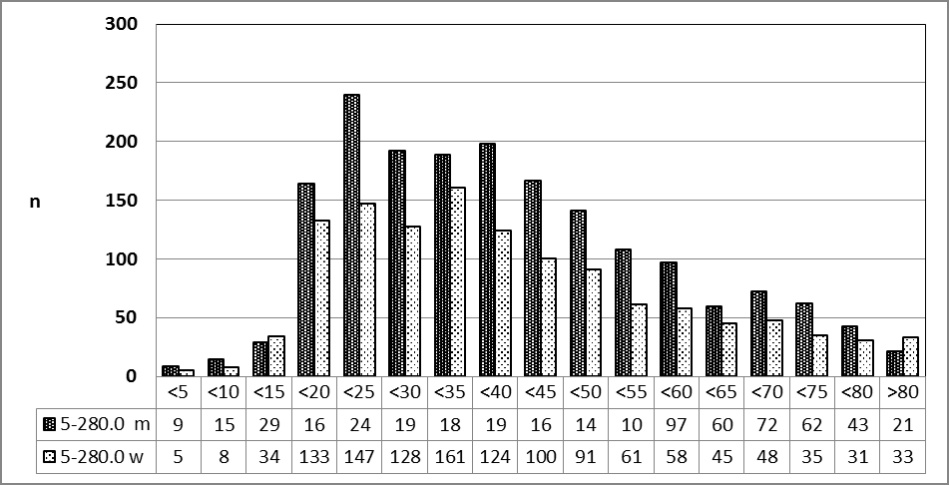
Prevalence of transoral incisional drainage (OPS code: 5-280.0) in 2010, detailed analysis The graphs of male and female patients indicate the prevalence (y-axis) according to age groups (x-axis). With the exception of younger patients (10–14 years of age) predominantly patients of male gender had undergone surgery. m = male; w = female

With the exception of patients between 10 and 14 years of age, patients of all age groups were predominantly of male gender. This contrasts to the finding for abscesstonsillectomy (Figure 23 (Fig. 23)).

##### 3.5.5.2 2013 [26]

**Figure 24:**
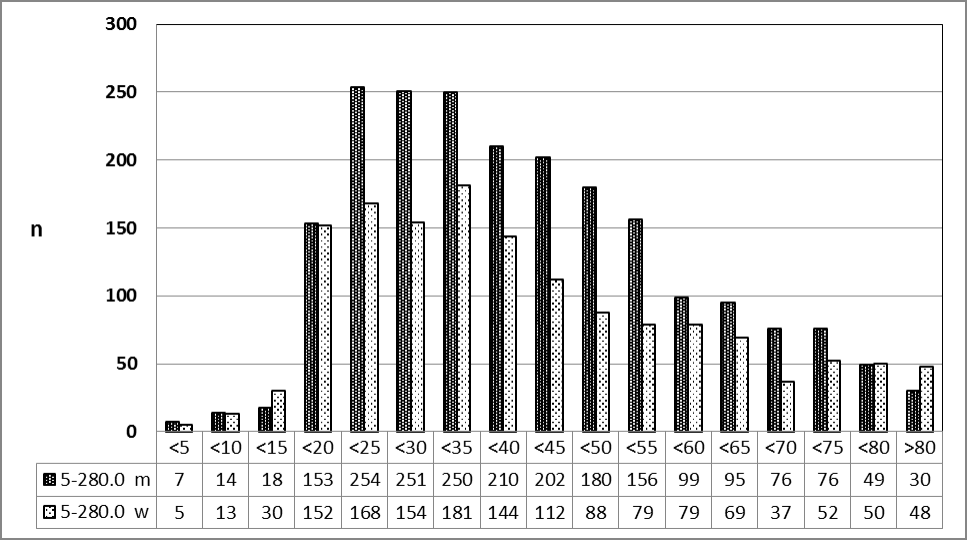
Prevalence of transoral incisional drainage (OPS code: 5-280.0) in 2013, detailed analysis The graphs of male and female patients indicate the prevalence (y-axis) according to age groups (x-axis). With the exception of younger patients (10–14 years of age) predominantly patients of male gender had undergone surgery. m = male; w = female

The same finding as described in 3.5.5.1 was registered in 2013 (Figure 24 (Fig. 24)).

#### 3.5.6 Incisional drainage and abscess tonsillectomy; age distribution

##### 3.5.6.1 Male patients in 2010 [26]

**Figure 25:**
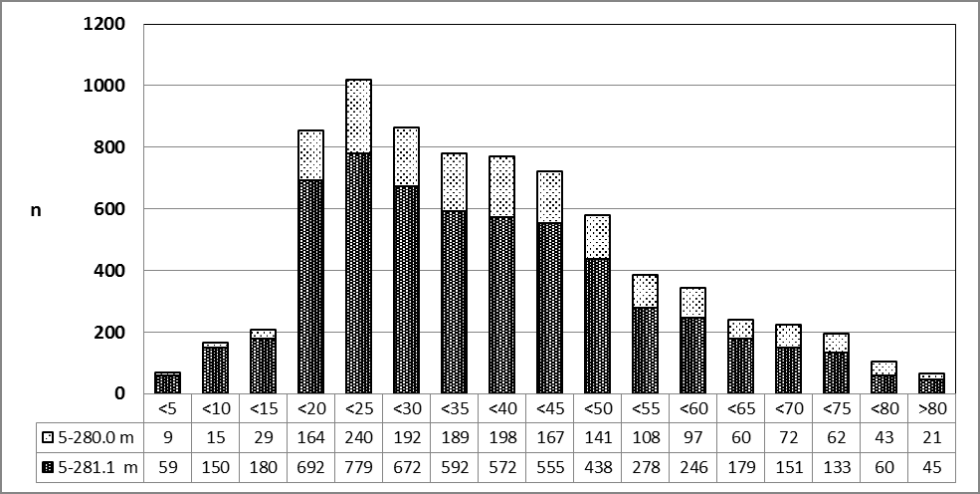
Prevalence of transoral incisional drainage (OPS code: 5-280.0) vs. abscesstonsillectomy (OPS code: 5-281.1) in male patients in 2010 The graphs indicate the prevalence (y-axis) of both interventions stratified by age groups (x-axis). Abscesstonsillectomy prevailed and incisional drainage was performed to a significant extent only after the age of 15 years. m = male; w = female

Abscesstonsillectomy prevailed as treatment modality with ID increasingly performed beyond the age of 15 (Figure 25 (Fig. 25)).

##### 3.5.6.2 Male patients in 2013 [26]

**Figure 26:**
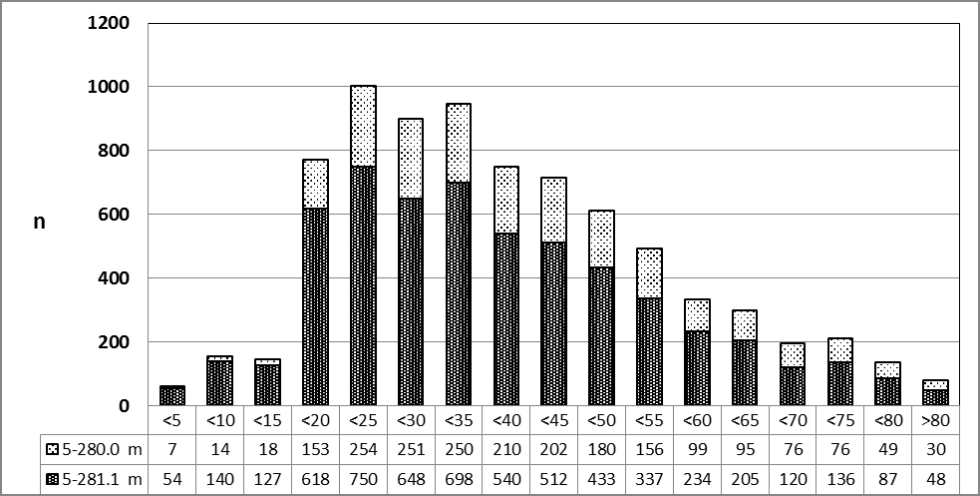
Frequency of transoral incisional drainage (OPS code: 5-280.0) vs. abscesstonsillectomy (OPS code: 5-281.1) in male patients in 2013 The graphs indicate the prevalence (y-axis) of both interventions stratified by age groups (x-axis). Abscesstonsillectomy prevailed and incisional drainage was performed to a significant extent only after the age of 15 years. m = male

The data of 2013 confirm the conclusions drawn for 2010 (Figure 26 (Fig. 26)).

##### 3.5.6.3 Female patients in 2010

**Figure 27:**
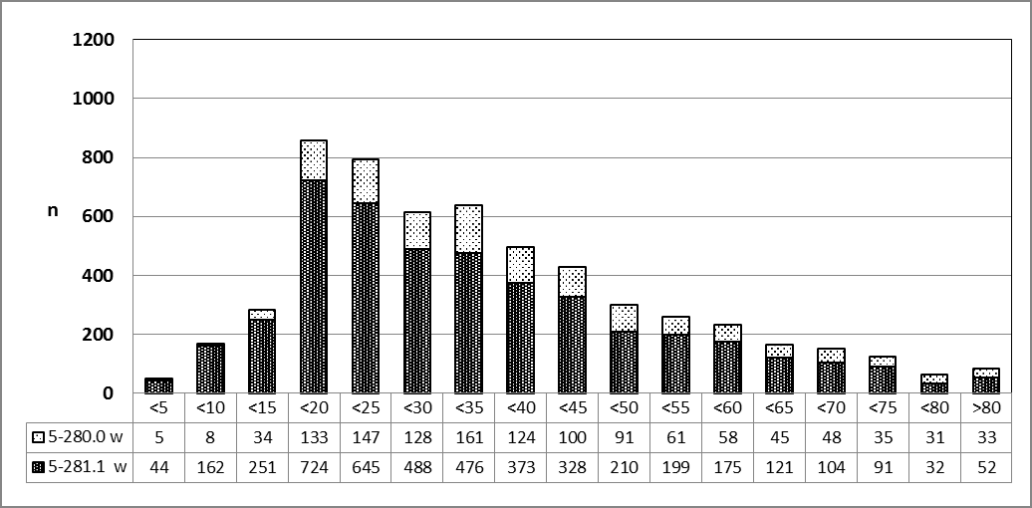
Frequency of transoral incisional drainage (OPS code: 5-280.0) vs*.* abscesstonsillectomy (OPS code: 5-281.1) in female patients in 2010 The graphs indicate the prevalence (y-axis) of both interventions stratified by age groups (x-axis). Abscesstonsillectomy prevailed and incisional drainage was performed to a significant extent only after the age of 15 years. w = female

Abscesstonsillectomy prevailed as treatment modality with incisional drainage increasingly performed beyond the age of 15 (Figure 27 (Fig. 27)).

##### 3.5.6.4 Female patients in 2013 [26]

**Figure 28:**
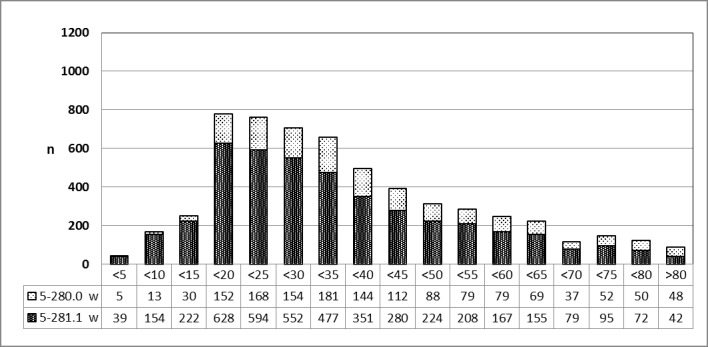
Frequency of transoral incisional drainage (OPS code: 5-280.0) vs. abscesstonsillectomy (OPS code: 5-281.1) in female patients in 2013 The graphs indicate the prevalence (y-axis) of both interventions stratified by age groups (x-axis). Abscesstonsillectomy prevailed and incisional drainage was performed to a significant extent only after the age of 15 years. w = female

The data of 2013 confirm the conclusions drawn for 2010 (Figure 28 (Fig. 28)). 

### 3.6 Post-tonsillectomy hemorrhage rate

A specialized database of the Federal Statistical Office was provided on request of the author. Due to the small number of cases (<500) in the group of elder patients (>65 years of age), post-tonsillectomy bleeding rates were not calculated for this age group. Moreover, other indications for TE are likely to indicate TE in elderly.

#### 3.6.1 Post-tonsillectomy hemorrhage rate in 2010 [26]

After 60,794 interventions in male patients (including: 5-280.0 (peri)tonsillar abscess; 5-281.0 TE with dissection technique; 5-281.1 abscesstonsillectomy; 5-281.2 radical transoral TE; 5-281.4 revision TE; 5-281.5 partial TE; 5-281.x others; 5-281.y not specified; 5-282.0 ATE with dissection technique; 5-282.x others; 5-282.y not specified), a bleeding rate of 7.04% was calculated 4,278 events. 

**Figure 29:**
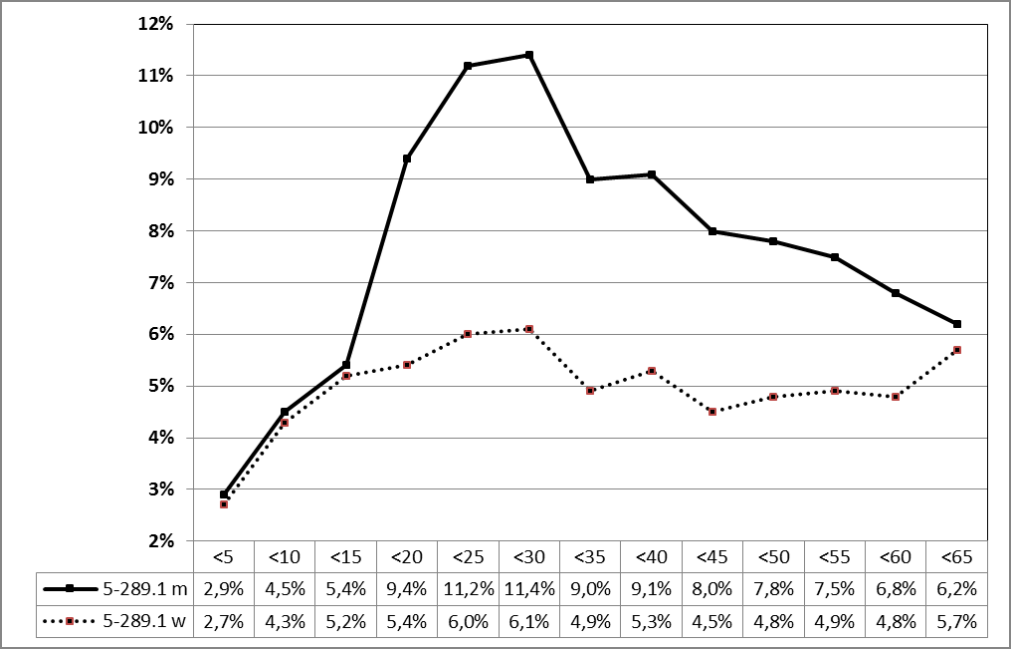
Rate of post-tonsillectomy hemorrhage in 2010 After 60,794 interventions in male patients (coded as explained in chapter 3.6.1), a postoperative bleeding rate of 7.04% was calculated vs. 5.02% after 70,292 interventions in female patients. The bleeding rate correlates with the age up to 15 years in boys and girls. Afterwards, the age-related hemorrhage rate increases only in male patients until the age of 30 years, and decreases later. In female patients, the postoperative bleeding rate was nearly unchanged after the age of 15 years. m = male; w = female

In female patients, a bleeding rate of 5.02% (3,530 events) was calculated after 70,292 procedures. Unfortunately, a limitation to bleeding events after elective TE (OPS code: 5-280.0) and thus a correlation is not exactly possible because bleeding complications might also have occurred after abscess, revision, or transpharyngeal TE as well as TOTO, but were assessed via the identical OPS code (5-281.9). The graphical illustration shows clearly the age-dependent phenomenon of post-tonsillectomy hemorrhage in male and female patients up to the age of 15, continually increasing only on male until the age of 30 years, while the rate remains almost unchanged in female patients (Figure 29 (Fig. 29)). 

#### 3.6.2 Post-tonsillectomy hemorrhage rate in 2013 [26]

**Figure 30:**
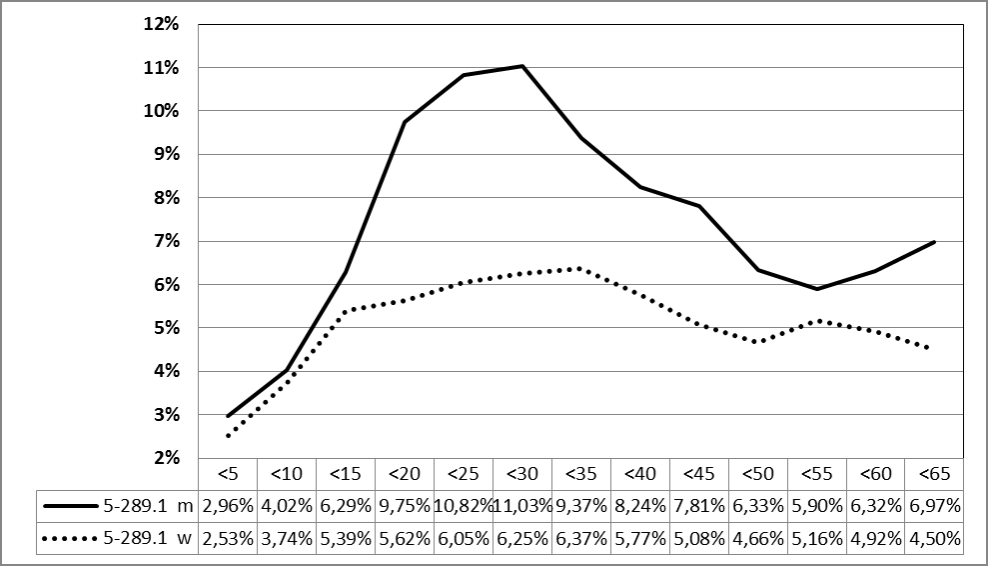
Rate of post-tonsillectomy hemorrhage in 2013 After 54,259 interventions in male patients (coded as explained in chapter 3.6.1), a postoperative bleeding rate of 6.99% was calculated vs. 5.11% after 60,376 interventions in female patients. The phenomena observed for 2010 can be reproduced in a nearly identical way. The bleeding rate correlates with the age up to 15 years in boys and girls. Afterwards, the age-related hemorrhage rate increases only in male patients until the age of 30 years, and decreases later. In female patients, the postoperative bleeding rate was nearly unchanged after the age of 15 years. m=male; w=female

After 54,259 interventions in male patients (coded as mentioned in 3.6.1), a bleeding rate of 6.99% was calculated based on 3,796 events. For female patients, a postoperative bleeding rate of 5.11% (3,088 events) was calculated after 60,376 procedures. The phenomena described for 2010 were confirmed including a pronounced age-related bleeding rate up to 15 years in female and 30 years in male patients (Figure 30 (Fig. 30)).

## 4 Discussion

### 4.1 Tonsillectomy

The role of TE within the public health system has been described in a previous paper, published in 2013 [27]. In 2010, ENT related interventions such as septoplasty, surgery of the nasal turbinates, TE, and neck dissection ranked among the 50 most frequently performed inpatient procedures in hospitals [21]. TE and ATE amounted to 128,133 interventions in 2010 which is equal to the numbers of cataract surgery (rank 24). Until the age of 14 years, ATE is the most frequently performed intervention in girls (12,094) and boys (13,724) [18]. A comprehensive analysis of national data in Germany was only recently published [10]. The analysis revealed variable regional differences in the prevalence of TE, ATE and TOTO according to an age and gender standardization. The 16 federal states were different with regard to the prevalence of TE in children and adolescents by factor 3, on the level of the 412 districts even by factor 8. This means that in some places only 1 of 900 children had undergone TE whereas in other regions 1 of 70 was scheduled for surgery. In relation to the indication “chronic tonsillitis” the difference was 12-fold and for “hypertrophy of the palatal tonsils” it was even 58-fold. However, a decrease in the prevalence was identified between 2007 and 2010 with an increasing number of TOTO procedures at the same time. This tendency was confirmed by the present study with a continuing decrease in the number of TEs and an increasing number of TOTOs in Germany [28]. 

As expected, the Bertelsmann study had triggered a great discussion about the current clinical practice in Germany. However, an attempt to compare the contemporary numbers with other nations is not obtainable from the literature. We therefore choosed to retrieve numbers from a database provided by the OECD (*Organization for Economic Cooperation and Development*) nations. This institution encompasses the following nations: Australia, Austria, Belgium, Canada, Chile, Czech Republic, Denmark, Estonia, Finland, France, Germany, Greece, Great Britain, Hungary, Ireland, Iceland, Israel, Italy, Japan, Korea, Luxemburg, Mexico, New Zealand, the Netherlands, Norway, Poland, Portugal, Turkey, and the USA. The data of these countries are open to online researches [29]. The respective calculations are possible by entering a key word such as *tonsillectomy, total number of procedures, total procedures per 100,000 population, % performed as inpatient cases, all countries, from 2005 to latest available date* [20].

It can be concluded from the OECD database, that TE is predominantly performed on an inpatient basis. The lowest rate of was found for the USA (3%), the highest rate Slovenia and Hungary (100%). In 2013, a total of 96.4% of the patients were hospitalized in Germany. Data from the Czech Republic, Greece, Iceland, Japan, Slovakia, Chile, and Estonia were not given. Based on calculations per 100,000 inhabitants, the lowest number of TE was registered in Mexico (23) and the highest number in the Netherlands (241.7) and the USA (254.4). Compared to the rates of those 29 countries (mean: 131.44; median: 111.6; standard deviation: 64.32), the rate in Germany is above the average (175.8 per 100,000 inhabitants). A comprehensive overview for 29 OECD nations is given in Table 8 (Tab. 8). However, the difference between the numbers provided by the Federal Statistical Office and the OECD remains to be clarified.

The scientific literature hardly ever provides the prevalence of TE and ATE. Some exceptions were found for Denmark: 7,000 [30], Scotland: 14,530 (sum from 2002–2005) and 3,605 (sum from 2006–2007) [31], Sweden: 10,000 [32], England with Wales: 19,250 [33], Italy: 44,000 [34] and 59,916 (2002) and 51,983 (2003) [11], France: 50,000 children [35], Great Britain: 78,000 [36] and 90,000 [37], and USA: 1,400,000 (1959) and 500,000 (1979) [38], 286,000 (1994) [39], [40] as well as 287,000 children (1996) [41] and 530,000 children [42], [43], [44] and 250,000 [45]. Sometimes also data on TE rates are found, as for example for Italy with a TE rate of 10.7 (2000), 10.5 (2002), 9.1 (2003), and 9.4 (2004) per 10,000 inhabitants [46]; for Australia with 64.0 per 10,000 children <10 years (2002–2004) [47] and New Zealand with 31 per 10,000 children ≤4 years and 43 per 10,000 children from 5–9 years (2003–2004) [47]. The TE rate (all types, but without abscesstonsillectomy) decreased from 13.34 (2010) to 10.90 per 10,000 inhabitants (2013) related to the total population.

### 4.2 Peritonsillar abscess

According to the present analysis, the rate for the diagnosis of “peritonsillar abscess” was 19.09 per 100,000 inhabitants and for the surgical procedures (abscesstonsillectomy and incisional drainage) 16.8 per 100,000 inhabitants in 2013. For the USA, Herzon calculated an annual rate of 30 per 100,000 [48]. For other countries, rates were given with 9 in Israel [49] up to 41 per 100,000 inhabitants in Denmark [50], [51], [52]. Love et al. reported an increasing incidence per year related to 100,000 inhabitants (1981–84: 9.8; 1990–92: 12.2; 2006-08: 28.7), but they had no adequate explanation for this phenomenon [53]. Klug et al. reported an age-related prevalence with the highest rate (167/100,000) in adolescents (15 to 19 years of age) [52] which is confirmed by the present study. Little et al. found an age-related change in the prevalence: 6.4 (<15 years), 24.7 (<45 years), 11.1 (<65 years), and 11.4 (>65 years) per 100,000 inhabitants per year [54]. For the age group between 14 and 21 years, Risberg et al. [55] identified the highest annual incidence (124/100,000). In some trials it becomes obvious that mainly male adolescents and adults are affected [56], [57], [58], with a ratio of 3:1 [53], [59]. There are other investigations that do not reveal a gender-related preponderance [49], [52], [53], [55], [59], [60], [61], [62], [63], [64], [65], [66], [67], [68]. The present study confirmed the finding, that patients younger than 19 years of age were predominantly female [53], [55], [58]. The present study also revealed that incisional drainage is an unusual procedure in patients younger than 15 years of age. However, incisional drainage was successfully performed by others even in children [69], [70], [71], [72], [73]. Unfortunately, the database of the Federal Statistic Office was not suitable to determine the number of interval tonsillectomies. It cannot be excluded that patients had undergone an elective TE after a certain period of time (then coded as 5-281.0) after an initial incisional drainage had been performed (coded as 5-280.0). The database also does not provide specific data for needle aspiration or antibiotic therapy alone for PTA [74], [75], [76], [77].

### 4.3 Tonsillotomy

The increasing number of TOTO in Germany was first described by the Bertelsmann Company [10] and is confirmed by the present study. However, the increasing number of TOTO procedures does not completely compensate the decrease in the prevalence of TE and ATE. Unfortunately, the database of the OECD countries does not provide any information concerning TOTO. The Austrian TE study (with an undetermined number of outpatient TOTOs) revealed a TOTO:TE ration of 1:3.48 in 2010. In Germany, the ratio was 1:13.6 in 2010 and 1:8.5 in 2013.

### 4.4 Postoperative bleeding rates

Since the Bertelsmann Study does not focus on postoperative bleeding rates, the rates were calculated for 2010, a year that was exhaustively analyzed, and compared to 2013 which is the most recent data provided by the Federal Statistical Office. In 2010 and 2013, a total of 14,692 bleedings had occurred after 245,721 interventions (5.98%). Postoperative bleedings were registered in 8,074 male patients after 115,053 interventions (7.02%) and in 6,618 female patients after 130,668 procedures (5.06%), respectively. 

A noticeably low rate of bleeding complications was reported by the “*National Prospective Tonsillectomy Act*” (NPTA) in Great Britain. Here, 1,197 bleeding events were registered after 33,291 interventions within the first 28 postoperative days (3.53%) of whom 318 required a surgical revision (0.94%). These data are in contrast to the much higher postoperative bleeding rates of the present study. Moreover, the bleeding rates were not related to age. However, gender was identified as a risk factor for post-tonsillectomy hemorrhage: the bleeding rate was 50% higher than in female patients (1.2% vs. 0.8% for bleedings requiring surgical revision; total rate: 3.7% vs. 3.4%).

In the *Austrian Tonsillectomy Study* (ATS) with 4,594 TE and ATE and 1,319 TOTO, with or without AT, 689 bleeding complications were registered after TE and ATE (15%) and 30 bleedings after TOTO, with or without AT (2.3%) [78]. A comparable amount of female and male patients were scheduled for TE (2,384 male vs. 2,210 female patients) which contrasts to the findings of the present study. In relation to the subpopulation of patients, who underwent TOTO, more male than female patients had undergone the operation (863 male vs. 456 female patients). For both interventions – TE and TOTO – postoperative bleeding was observed more often in male patients (390 vs. 299; 17 vs. 13). Bleedings of all intensities were related to age in TE and TOTO in this study, but in the aforementioned NPTA as well as in the ATS only 3 age categories were compared (6; 6–15; >15 years). According to the data of the present study, it appears advisable to use the age of 20 years as cut-off value since beyond that age a decreasing post-tonsillectomy hemorrhage rate is likely to occur. Bleeding events requiring surgical revision after TE were registered in 154 male (6.3%) and 95 female patients (4.3%). For TOTO, more female patients required surgical revision after surgery (1.3% vs. 0.7%).

### 4.5 Phenomena

1. Decreasing frequency of diagnosis
  Considering the frequency of diagnosis of “chronic tonsillitis”, “hypertrophy of the palatal tonsils” as well as “hypertrophy of the palatal tonsils with hypertrophy of the pharyngeal tonsil”, a decrease by factor 2.16, 1.37, and 1.53 was found for Germany between 2000 and 2013 which cannot be explained scientifically. This phenomenon has no justification in the population development in the same period. Moreover, no scientific argument is obtainable to explain the higher prevalence of “chronic tonsillitis” in female patients and an equal prevalence of hypertrophy of the tonsils/adenoids in boys and girls. An imbalanced gender ratio of the population does not exist, as shown in Table 9 (Tab. 9) [79].
2. Decreasing number of cases
  Between 2005 and 2013, the number of TE in Germany decreased (without simultaneous AT) by factor 1.12. In female patients, this factor was higher (1.18) compared to factor 1.06 in male patients. The detailed analysis shows a significant decrease of the case numbers especially in the age group of 5–10 years as well as 15–20 years. This aspect can only be described as a phenomenon, but not explained scientifically.
3. Female preponderance
  In the present study the male to female ratio was in total 1:1.29 for TE which differs to Great Britain (1:1.49) and in Austria (1:0.82) [79], [80]. The detailed analysis of two years, 2010 and 2013, revealed a striking difference in the prevalence of the TE and ATE between female and male patients, especially in the age group younger than 20 years. A twofold higher rate in female patients was determined in young adults (15 to 20 years of age) who had undergone TE. For ATE, the difference was even a threefold higher rate for the same age group which cannot be explained scientifically. This phenomenon has been described earlier for a population (n=10,095) of three German ENT departments with a significant different prevalence of TE particularly in younger patients (16 to 20 years of age) [81]. The authors suggested a specific psychic constellation in female patients at that particular age, but this hypothesis cannot be clarified retrospectively. However, the phenomenon described by Spicker and Schultz-Coulon was confirmed by the present study (n=627,874). It is noteworthy to repeat, that analysis of data retrieved from the Federal Statistical Office database could exclude an imbalanced gender distribution in the German population.
4. PTA
  The sudden increase of the prevalence at an age of 15 years with a decrease at an age of 25 years cannot be explained scientifically. Moreover, the preponderance of female gender only at an age between 10 to 15 years remains a conundrum.
5. Post-tonsillectomy hemorrhage
  Several risk factors for bleeding complications after TE and ATE have been described, such as male gender [82], [83], [84], [85], [86], [87], [88], [89], [90]. To the best of our knowledge, a different gender-specific and age-related post-tonsillectomy hemorrhage rate has never been described before.

### 4.6 Limitations

Different entities may be summarized with a single ICD-10 code, impeding a differentiated analysis of the data provided by the Federal Statistical Office. “J35” is a good example for this problem (see 3.1.2) and may explain the two peaks in the prevalence related to age. “J35” includes “chronic tonsillitis” (ICD-10: J35.0), “hypertrophy of the palatal tonsils” (ICD-10: J35.1), “hypertrophy of the pharyngeal tonsil” (ICD-10: J35.2), “hypertrophy of the palatal tonsils and the pharyngeal tonsil”, and “others”, respectively. 

The data of all procedures performed on an outpatient are not registered by the Federal Statistical Office or any other institution. This unknown number of procedures was therefore not included in the present study.

The concept of interval TE (elective TE after previous transoral incisional drainage) for PTA cannot be decoded because a coherent depiction of the single cases is virtually impossible.

### 4.7 Summary

The prevalence of chronic diseases of the palatal tonsils as well as the absolute and relative frequency of tonsillectomy has clearly decreased within the investigated time period, predominantly in female patients. In contrast, the number of TOTOs has increased continually, particularly in in male patients. Numbers and rates of different treatment modalities for PTA have not considerably changed. However, it appears as if incision and drainage has become a more popular intervention but abscesstonsillectomy clearly prevails. Regardless the indication for surgery, bleeding complications had occurred preferably in male patients, especially in adolescents and young adults.

## Notes

### Competing interests

The author declares that he has no competing interests.

## Figures and Tables

**Table 1:**
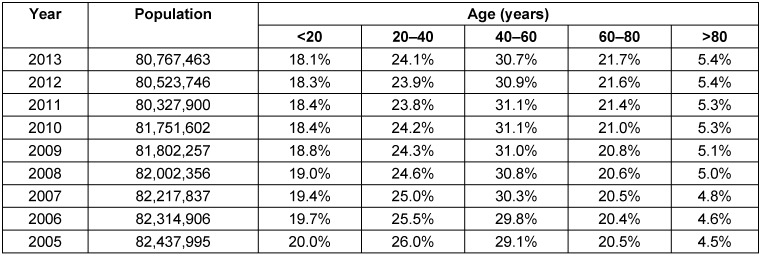
Population and age structure in Germany [23]

**Table 2:**
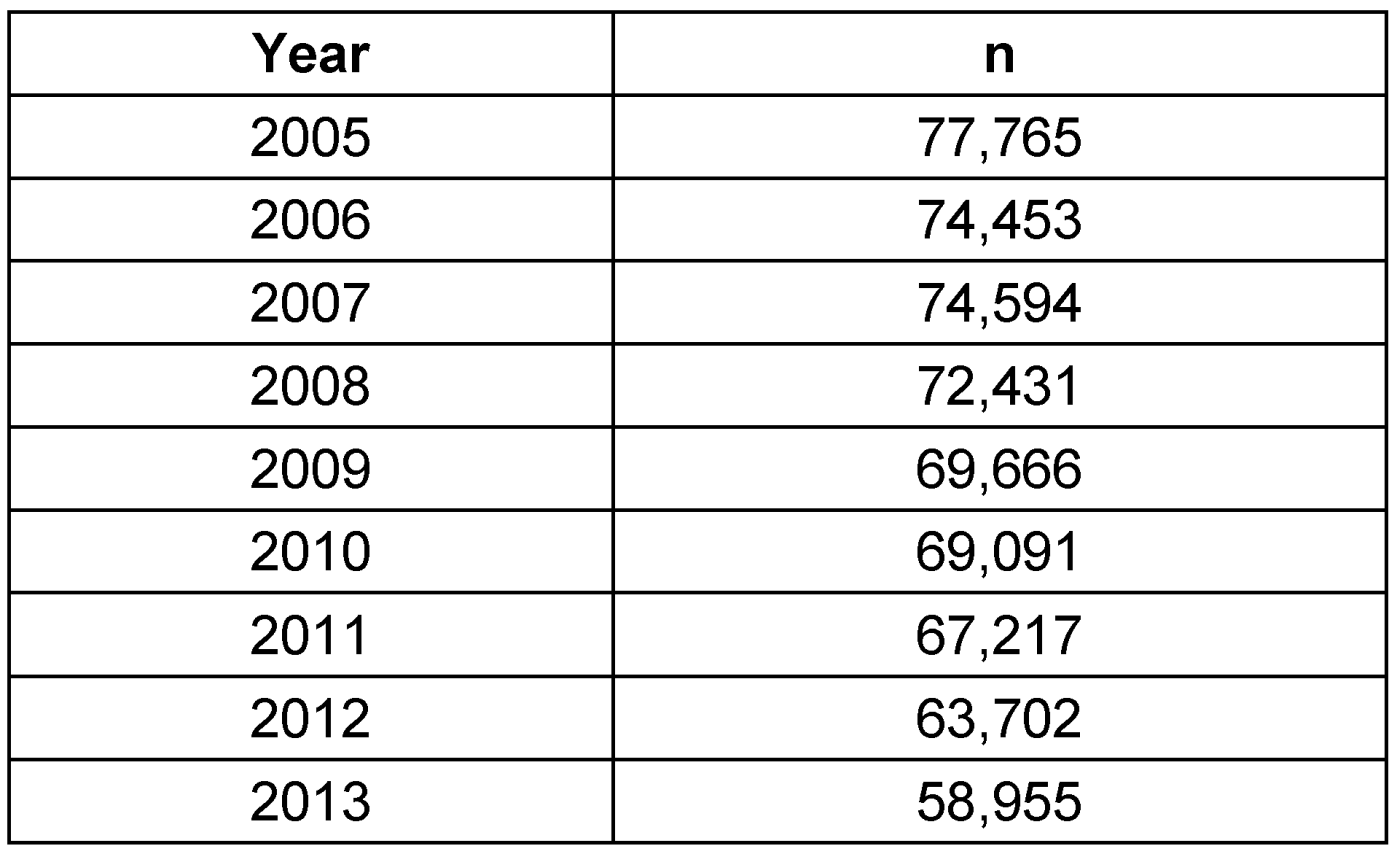
Tonsillectomy without adenoidectomy (OPS code: 5-281.0). Number of procedures per year (2005–2013)

**Table 3:**
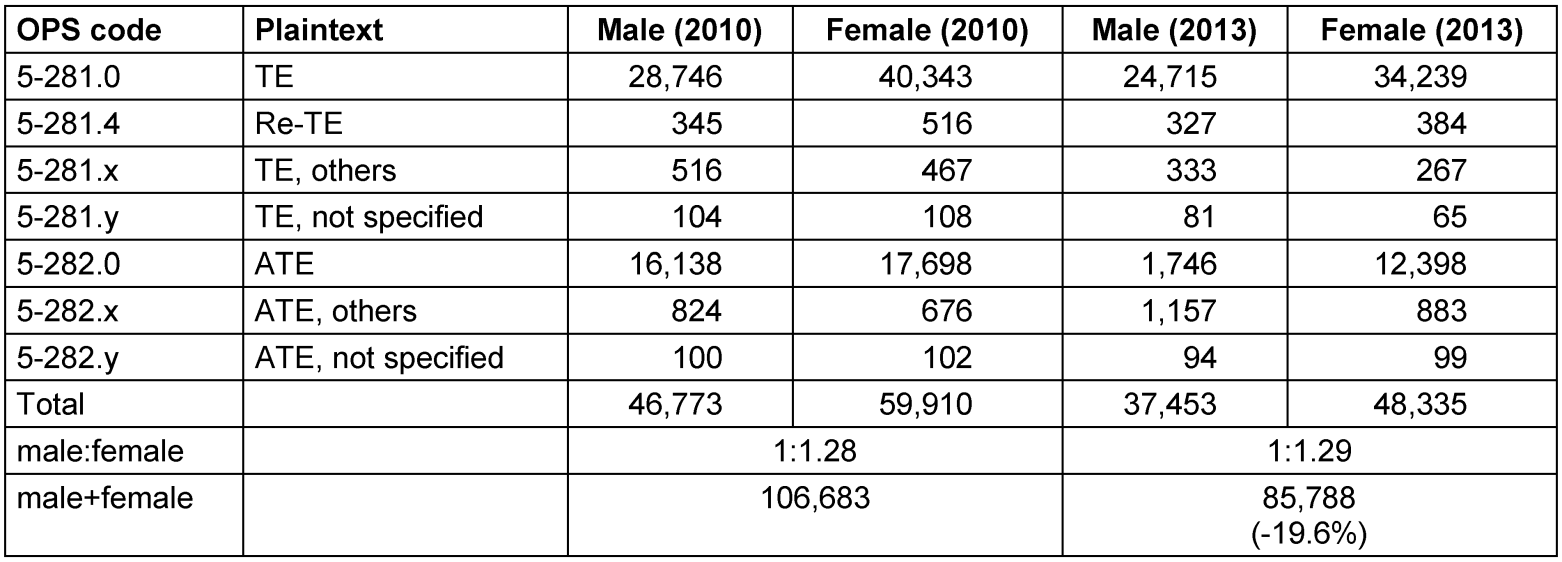
Prevalence of tonsillectomy in patients <20 years (2010 vs. 2013) The procedures were predominantly performed in female patients. The number of interventions had decreased by 19.6% within 3 years. (ATE = adenotonsillectomy; TE = tonsillectomy; Re-TE = revision tonsillectomy)

**Table 4:**
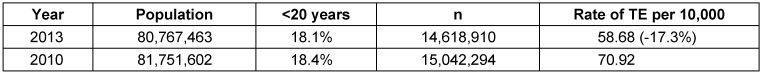
Rate of tonsillectomy in patients younger than 20 years Based on the data of Table 3, the rates of tonsillectomy for patients younger than 20 years could be calculated for 2010 and 2013 with relation to the population statistics. Within 3 years, the rate had decreased by 17.3%.

**Table 5:**
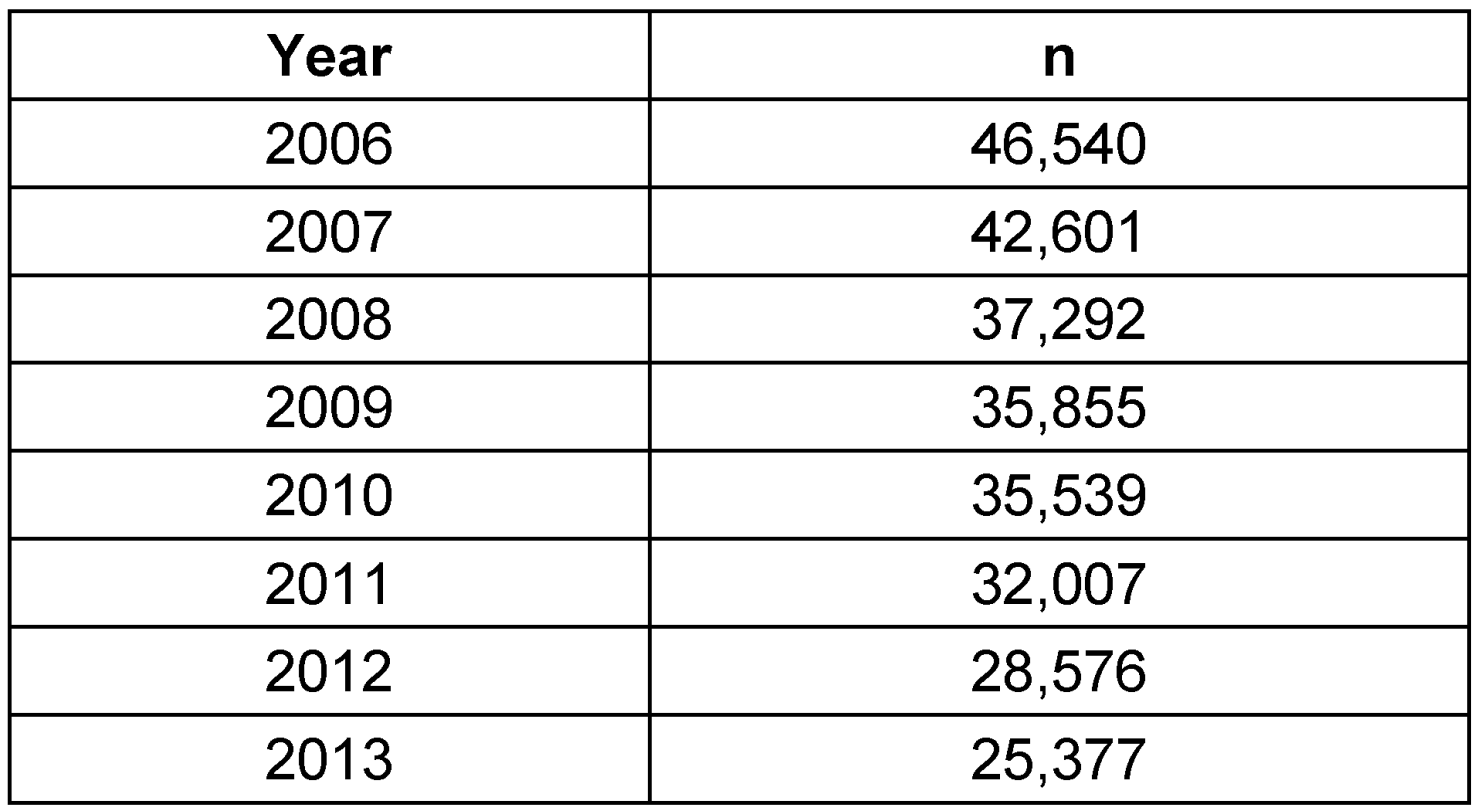
Adenotonsillectomy (OPS code: 5-282) Between 2006 and 2013, a total of 283,787 interventions were performed, revealing a decreasing yearly prevalence.

**Table 6:**
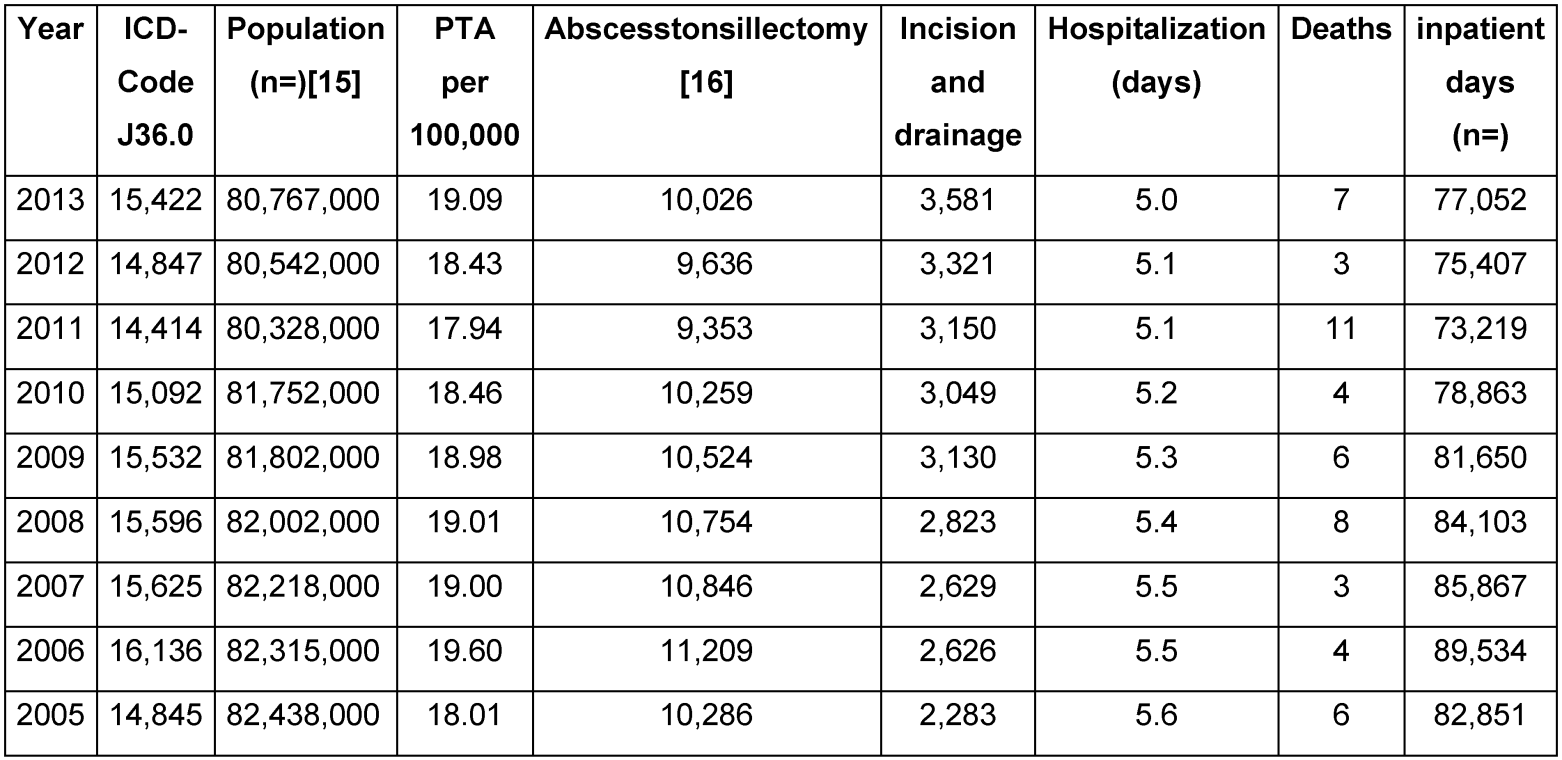
Peritonsillar abscess (ICD-10: J36). Prevalence, rates, and burden of health. PTA = peritonsillar abscess

**Table 7:**
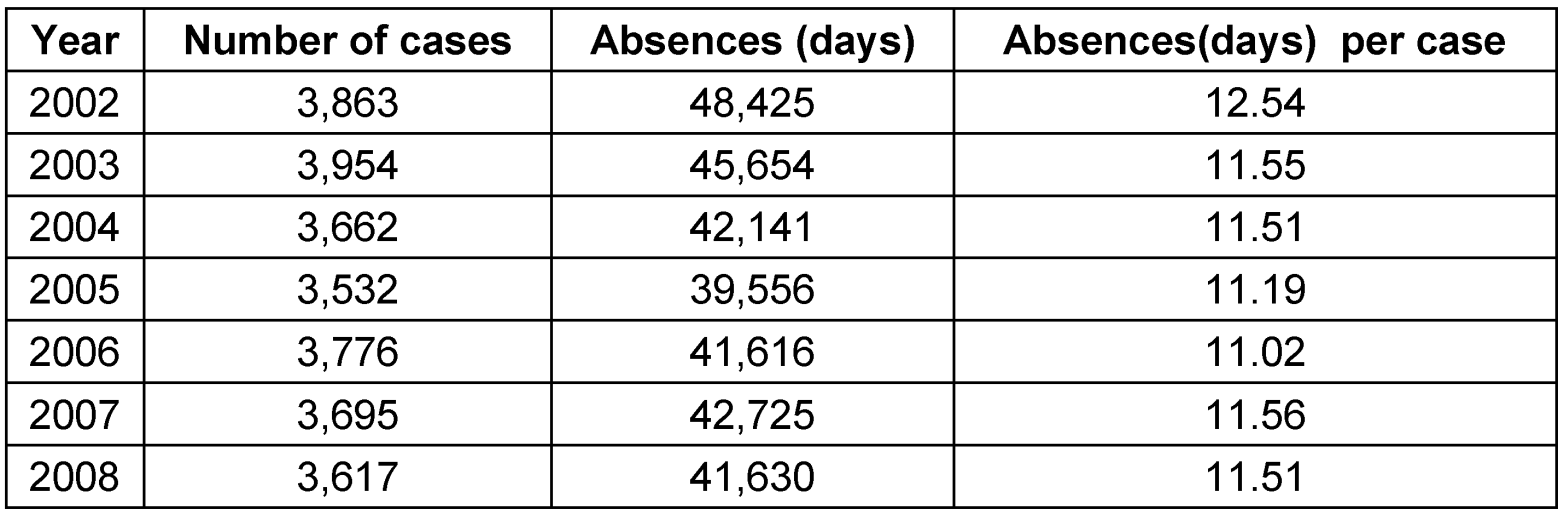
Absences from work due to peritonsillar abscess, analysis per year. The table shows a decreasing tendency of the prevalence, the number of days of absences and number of days of absences per case between 2002 and 2008.

**Table 8:**
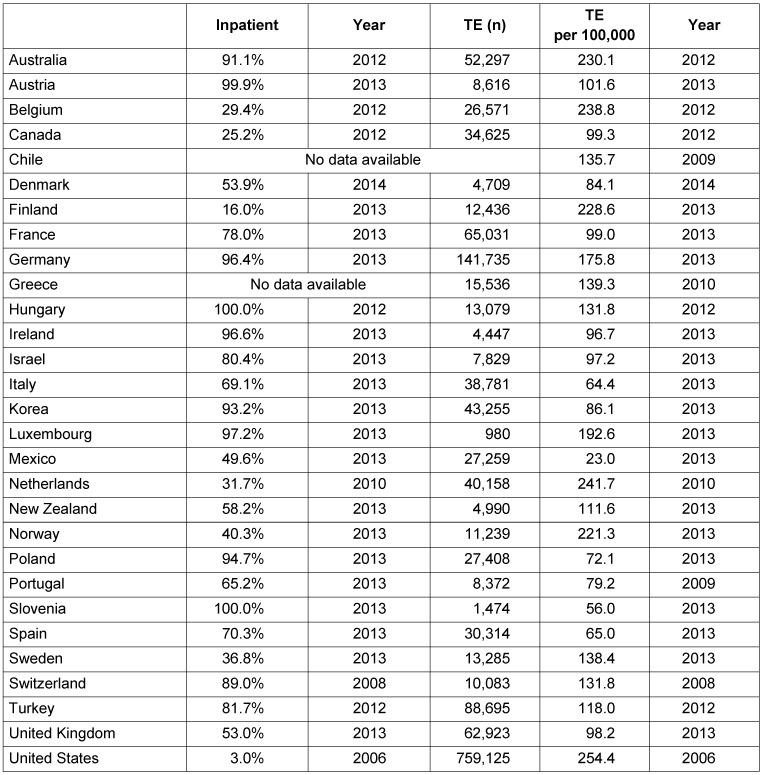
Tonsillectomy in OECD member countries

**Table 9:**
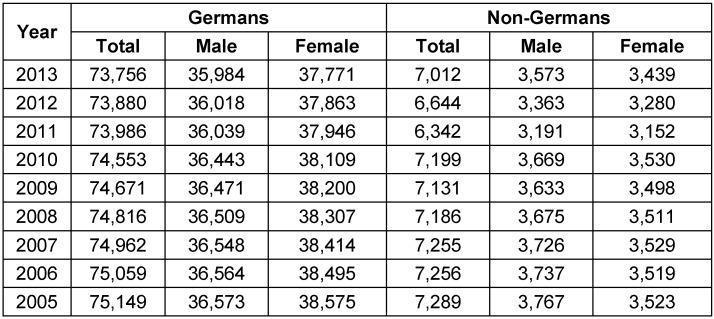
Population structure in Germany (in thousands)
